# DWORF expression is reduced in a large animal model of Duchenne muscular dystrophy

**DOI:** 10.1242/dmm.052285

**Published:** 2025-06-25

**Authors:** Aaron M. Gibson, Xiufang Pan, Omar Brito-Estrada, James A. Teixeira, Lauren K. Carl, Yongping Yue, Michael L. Kamradt, Matthew J. Burke, Caris A. Wadding-Lee, Gang Yao, Roland W. Herzog, Dongsheng Duan, Catherine A. Makarewich

**Affiliations:** ^1^ The Heart Institute, Division of Molecular Cardiovascular Biology, Cincinnati Children's Hospital Medical Center, Cincinnati, OH 45229, USA; ^2^ Department of Molecular Microbiology and Immunology, School of Medicine, The University of Missouri, Columbia, MO 65212, USA; ^3^ Molecular and Developmental Biology Graduate Program, University of Cincinnati College of Medicine, Cincinnati, OH 45267, USA; ^4^ Department of Chemical and Biomedical Engineering, College of Engineering, The University of Missouri, Columbia, MO 65212, USA; ^5^ Department of Pediatrics, Herman B Wells Center for Pediatric Research, Indiana University, Indianapolis, IN 46202, USA; ^6^ Department of Neurology, School of Medicine, The University of Missouri, Columbia, MO 65212, USA; ^7^ Department of Biomedical Sciences, College of Veterinary Medicine, The University of Missouri, Columbia, MO 65212, USA; ^8^ Department of Pediatrics, University of Cincinnati College of Medicine, Cincinnati, OH 45229, USA

**Keywords:** Duchenne muscular dystrophy, *DMD*, DWORF, SERCA2a, Canine model

## Abstract

Duchenne muscular dystrophy (DMD) is a lethal muscle-wasting disease driven by cytosolic calcium overload, which leads to muscle degeneration. Sarco/endoplasmic reticulum calcium ATPase (SERCA), a key regulator of cytosolic calcium levels, exhibits reduced activity in animal models of DMD and human patients. Dwarf open reading frame (DWORF), a positive SERCA regulator, is downregulated in *mdx* DMD mice, and adeno-associated virus-mediated DWORF overexpression has been shown to ameliorate DMD cardiomyopathy. The canine DMD model provides a crucial bridge for translating findings from mice to humans. To investigate DWORF expression in this model, we developed a canine-specific anti-DWORF antibody, as the existing murine antibody is ineffective. This antibody detected DWORF in human, pig, cat and rabbit muscle, but not in mouse muscle. DWORF was absent in muscle tissues of neonatal normal dogs but highly expressed in those of adult dogs. In DMD-affected dogs aged 8 months or older, DWORF expression was significantly reduced in both cardiac and skeletal muscle. This study establishes a foundation for evaluating DWORF-based gene therapy in the canine DMD model, advancing the potential for clinical translation.

## Teaser

**Summary:** This study developed an anti-DWORF antibody capable of recognizing the canine protein and revealed reduced DWORF expression in Duchenne muscular dystrophy (DMD) dogs, thus laying the groundwork for DWORF-based gene therapy to treat DMD.

## INTRODUCTION

Duchenne muscular dystrophy (DMD) is the most common inherited muscle-wasting disease in boys and is caused by null mutations in the *DMD* gene (Duan et al., 2021). Dystrophin is a critical structural protein that serves as a core component of the dystrophin–glycoprotein complex (DGC), linking the actin cytoskeleton to the extracellular matrix. This connection stabilizes the cell membrane and mediates essential signaling events, including mechanical force transduction. Dystrophin deficiency leads to membrane instability and rupture, causing cytosolic calcium influx, mitochondrial calcium overload and the direct activation of calcium-dependent proteases, resulting in myocyte necrosis, which in turn triggers an inflammatory response leading to tissue fibrosis (Burr and Molkentin, 2015; Law et al., 2020; Mareedu et al., 2021a; Rahimov and Kunkel, 2013). In the heart, this process contributes to cardiomyocyte death, fibrosis, ventricular dilation and reduced pump function, while in skeletal muscle, progressive muscle weakness occurs owing to similar pathological mechanisms.

Calcium plays a critical role in cardiac and skeletal muscle excitation–contraction coupling through its direct interaction with myofilament proteins, and calcium transport from the cytoplasm to the sarcoplasmic reticulum (SR) drives muscle relaxation (Bers, 2002). Calcium also serves as an essential second messenger in numerous cellular signaling pathways, making its precise regulation crucial for cellular health. The SR is the primary intracellular calcium storage site, and its calcium loading is mediated by the sarco/endoplasmic reticulum calcium-ATPase [SERCA; SERCA1 (also known as ATP2A1) in skeletal muscle and SERCA2a (also known as ATP2A2) in the heart]. Although the primary defects in DMD arise from disruption of the membrane-stabilizing DGC, studies in genetically modified mice with artificially elevated intracellular calcium levels have demonstrated that cytosolic calcium overload is the final common pathway leading to myocyte necrosis, muscle degeneration and cardiac dilation in dystrophic disease (Burr et al., 2014; Goonasekera et al., 2014; Millay et al., 2009). Notably, reducing cytosolic calcium levels by overexpressing or activating SERCA (Goonasekera et al., 2011; Kodippili et al., 2024; Mazala et al., 2015; Morales et al., 2023; Morine et al., 2010; Nogami et al., 2021; Shin et al., 2011; Wasala et al., 2020) or downregulating the SERCA inhibitor sarcolipin (SLN) (Balakrishnan et al., 2022; Mareedu et al., 2021b; Voit et al., 2017) has been shown to reduce myocyte necrosis and attenuate the progression of dystrophic disease. These findings underscore the therapeutic potential of enhancing SERCA activity in DMD and other dystrophic diseases.

The SERCA-activating microprotein dwarf open reading frame (DWORF; also known as STRIT1) directly binds SERCA and increases its calcium affinity and maximal calcium-transport activity (Nelson et al., 2016). In mice, cardiomyocyte-specific overexpression of DWORF via transgene or adeno-associated virus (AAV) increased SERCA activity, calcium cycling and myocyte contractility, and was cardioprotective in mouse models of heart failure (myocardial infarction) and dilated cardiomyopathy (*Csrp3* gene deletion) (Makarewich et al., 2020, 2018; Nelson et al., 2016). Most recently, we demonstrated that AAV-mediated overexpression of DWORF restored SERCA function and mitigated heart disease in a mouse model of Duchenne dilated cardiomyopathy (Morales et al., 2023). Furthermore, reduced DWORF expression in human heart failure and animal models of cardiac and skeletal muscle disease suggests that DWORF deficiency contributes directly to calcium dysregulation and disease pathogenesis (Makarewich et al., 2020, 2018; Morales et al., 2024, 2023; Nelson et al., 2016).

Although many mouse models, including the commonly used *mdx* model, have been developed to study DMD pathogenesis and potential therapies, these models fail to replicate several key aspects of the human disease (Yucel et al., 2018). To address this limitation, the canine DMD model has emerged as an invaluable translational tool, closely mimicking the severity and progression of human DMD (Duan, 2015; Kornegay, 2017; McGreevy et al., 2015). Unlike murine models, dogs exhibit more severe and human-like disease phenotypes, including progressive muscle weakness, respiratory decline and cardiomyopathy. Investigating DWORF expression in this model is crucial for understanding its role in DMD pathogenesis across species and evaluating its therapeutic potential. However, current tools for studying DWORF protein expression are limited to a single custom antibody designed for the mouse DWORF sequence, which cannot detect the canine protein. To overcome this challenge, we developed a new custom antibody specific to canine DWORF and demonstrated that DWORF expression is significantly reduced in the skeletal muscle and heart of adult DMD dogs. This study represents the first characterization of DWORF protein expression in a large-animal model of DMD and establishes a foundation for preclinical testing of DWORF-based therapies.

## RESULTS

### Mouse anti-DWORF antibody does not recognize canine DWORF protein

**Fig. 1.**
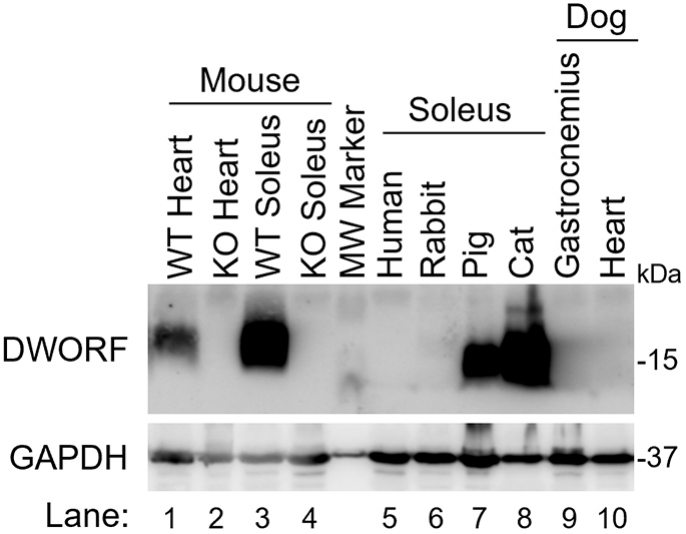
**Evaluation of the mouse anti-DWORF antibody.** Cross-species evaluation of the mouse anti-DWORF antibody by western blot analysis. The following muscle tissues from adult non-diseased (normal) animals were examined (lane number indicated in parentheses): (1) wild-type mouse heart, (2) DWORF knockout mouse heart, (3) wild-type mouse soleus, (4) DWORF knockout mouse soleus, (5) human soleus, (6) rabbit soleus, (7) pig soleus, (8) cat soleus, (9) dog gastrocnemius and (10) dog heart. KO, knockout; kDa, molecular mass in kilo Dalton; WT, wild type.

DWORF is a transmembrane microprotein that embeds in the SR membrane and directly interacts with SERCA to exert its potent SERCA-activating effects. We previously developed a custom polyclonal antibody targeting the cytosolic N-terminus of the mouse DWORF protein (Nelson et al., 2016) and validated its use for murine DWORF detection via western blotting (Makarewich et al., 2020, 2018; Morales et al., 2023; Nelson et al., 2016). To assess cross-reactivity with DWORF in other species, we performed western blot analysis of proteins isolated from muscle tissues of humans, rabbits, pigs, cats and dogs (Fig. 1). The antibody successfully detected DWORF protein in mouse, pig and cat tissues, but not in human, rabbit or dog tissues.

### Development of dog-specific polyclonal anti-DWORF antibody

**Fig. 2.**
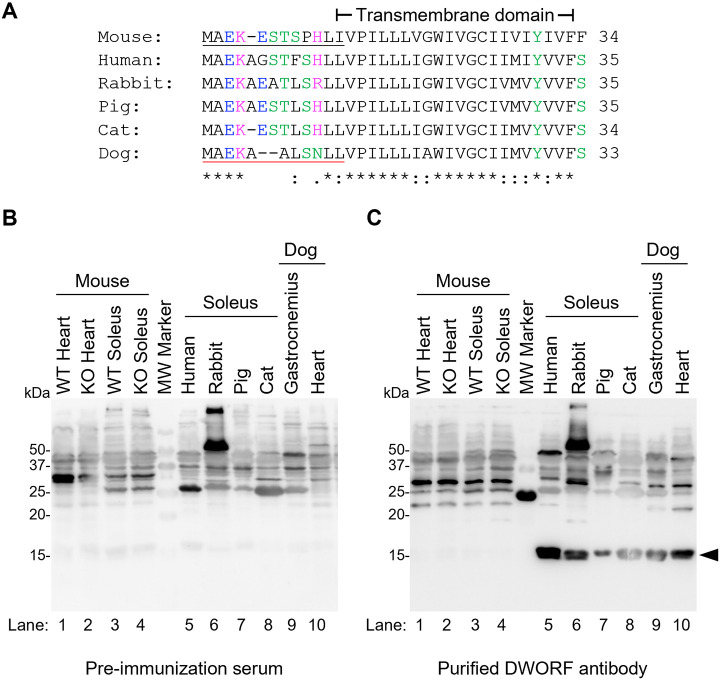
**Validation of a canine DWORF antibody.** (A) Multi-species alignment of DWORF amino acid sequences performed by Clustal Omega (see Materials and Methods). The epitopes used to generate species-specific antibodies are underlined (mouse, black; dog, red). The symbols below the alignment indicate the degree of conservation of each amino acid: asterisks, fully-conserved residues; colons, strongly similar properties; periods, weakly similar properties. Each amino acid is colored to depict its properties (black, non-polar; green, polar neutral; pink, polar basic; blue, polar acidic). (B,C) Western blot analysis of heart and skeletal muscle from the indicated species using pre-immunized rabbit serum (B) or the purified canine anti-DWORF antibody (C). The following muscle tissues from adult non-diseased (normal) animals were examined (lane number indicated in parentheses): (1) wild-type mouse heart, (2) DWORF knockout mouse heart, (3) wild-type mouse soleus, (4) DWORF knockout mouse soleus, (5) human soleus, (6) rabbit soleus, (7) pig soleus, (8) cat soleus, (9) dog gastrocnemius and (10) dog heart. Arrowhead indicates DWORF.

The murine anti-DWORF antibody targets an N-terminal epitope of the mouse DWORF sequence (MAEKESTSPHLI) (Fig. 2A) and recognizes both the 34- and 35-amino acid isoforms of the protein. The 35-amino acid isoform includes an additional alanine (underlined) at the exon 2 splice site (MAEKAESTSPHLI). Although the C-terminal transmembrane domain of DWORF is highly conserved across species, its N-terminus shows poor conservation between mouse and dog. To develop a dog-specific polyclonal DWORF antibody, rabbits were immunized with the N-terminal region of canine DWORF (MAEKAALSNLL) (Fig. 2A). The resulting sera were collected and affinity purified against the peptide immunogen. Preimmunization rabbit serum showed a weak, nonspecific band near the predicted molecular mass of DWORF in all muscle samples tested (Fig. 2B). The presence of this weak band in heart and soleus tissue from DWORF knockout mice, which do not express DWORF, confirmed that it is a nonspecific band. Conversely, the purified dog-specific DWORF antibody produced a strong band at the predicted molecular mass in skeletal muscle from humans, rabbits, pigs, cats and dogs, as well as in heart tissue from dogs. A DWORF-positive band was not detected in normal or DWORF knockout mouse muscle tissues (Fig. 2C), indicating that the canine DWORF antibody does not recognize the mouse DWORF protein.

### DWORF is expressed in skeletal muscle and hearts of adult dogs

To assess the temporal expression pattern of DWORF in canine tissues, we analyzed protein levels in skeletal and cardiac muscles from dogs of varying ages. Specifically, we examined the extensor carpi ulnaris (ECU), diaphragm, left ventricle (LV) and right atrium (RA) using western blot analysis with the dog-specific anti-DWORF antibody. The diaphragm was included owing to it being severely affected in DMD (Duan et al., 2021), while the ECU was selected for its established use in evaluating gene therapy targets in canine models (Hakim et al., 2021; Kodippili et al., 2024, 2018; Shin et al., 2013; Yang et al., 2012). We analyzed tissues from neonatal (<0.4-month-old) and adult (8- to 13-month-old and 30- to 55-month-old) dogs (Table 1). This approach allowed us to examine developmental and age-related changes in DWORF expression across key skeletal and cardiac tissues.

**Table 1.** Dog sample information

Age range	Dog ID#	Genotype	Sex	Age (months)	RA	LV	Diaphragm	ECU	Gas
<0.4 months	Dog #1	Normal	M	0.03	No	Yes	Yes	Yes	No
	Dog #2	Normal	M	0.07	No	Yes	Yes	Yes	No
	Dog #3	Normal	M	0.13	No	Yes	Yes	Yes	No
	Dog #4	Normal	M	0.17	Yes	Yes	Yes	Yes	No
	Dog #5	Normal	M	0.23	No	Yes	Yes	Yes	No
	Dog #6	Affected	M	0.03	Yes	Yes	Yes	No	No
	Dog #7	Affected	M	0.16	Yes	Yes	Yes	Yes	No
	Dog #8	Affected	F	0.30	Yes	Yes	Yes	Yes	No
	Dog #9	Affected	M	0.36	Yes	Yes	Yes	Yes	No
8-13 months	Dog #10	Normal	F	8.61	Yes	Yes	No	No	No
	Dog #11	Normal	F	8.71	Yes	Yes	No	No	No
	Dog #12	Normal	M	12.30	Yes	Yes	Yes	Yes	Yes
	Dog #13	Normal	M	12.56	Yes	Yes	Yes	Yes	No
	Dog #14	Normal	M	12.76	Yes	Yes	Yes	Yes	No
	Dog #15	Normal	M	12.92	Yes	Yes	Yes	Yes	No
	Dog #16	Normal	M	13.28	Yes	Yes	Yes	Yes	No
	Dog #17	Affected	M	8.02	Yes	Yes	No	No	No
	Dog #18	Affected	F	8.78	Yes	Yes	Yes	Yes	No
	Dog #19	Affected	M	8.91	Yes	Yes	Yes	Yes	No
	Dog #20	Affected	F	8.98	Yes	Yes	Yes	No	No
	Dog #21	Affected	M	12.46	Yes	Yes	Yes	Yes	No
	Dog #22	Affected	M	13.05	Yes	Yes	Yes	Yes	No
30-55 months	Dog #23	Normal	M	29.92	Yes	Yes	Yes	Yes	No
	Dog #24	Normal	M	30.61	Yes	Yes	Yes	Yes	No
	Dog #25	Normal	M	31.50	Yes	Yes	Yes	Yes	No
	Dog #26	Normal	M	36.82	Yes	Yes	No	No	No
	Dog #27	Normal	M	37.38	Yes	Yes	No	No	No
	Dog #28	Normal	M	45.47	Yes	Yes	Yes	Yes	No
	Dog #29	Normal	M	47.84	Yes	Yes	No	No	No
	Dog #30	Normal	F	55.43	Yes	Yes	Yes	Yes	No
	Dog #31	Affected	F	30.81	Yes	No	No	No	No
	Dog #32	Affected	F	30.84	Yes	Yes	No	No	No
	Dog #33	Affected	M	33.04	Yes	Yes	No	No	No
	Dog #34	Affected	M	33.30	Yes	Yes	Yes	Yes	No
	Dog #35	Affected	M	34.26	Yes	Yes	Yes	Yes	No
	Dog #36	Affected	M	34.82	Yes	Yes	Yes	Yes	No
	Dog #37	Affected	M	38.70	Yes	Yes	Yes	Yes	No
	Dog #38	Affected	M	38.70	No	Yes	No	No	No
	Dog #39	Affected	M	41.62	Yes	Yes	No	No	No
	Dog #40	Affected	M	41.79	Yes	Yes	No	No	No
	Dog #41	Affected	M	41.95	Yes	Yes	No	No	No
	Dog #42	Affected	M	44.81	Yes	Yes	Yes	Yes	No
	Dog #43	Affected	M	46.06	Yes	Yes	No	No	No

ECU, extensor carpi ulnaris; Gas, gastrocnemius; LV, left ventricle; RA, right atrium.

**Fig. 3.**
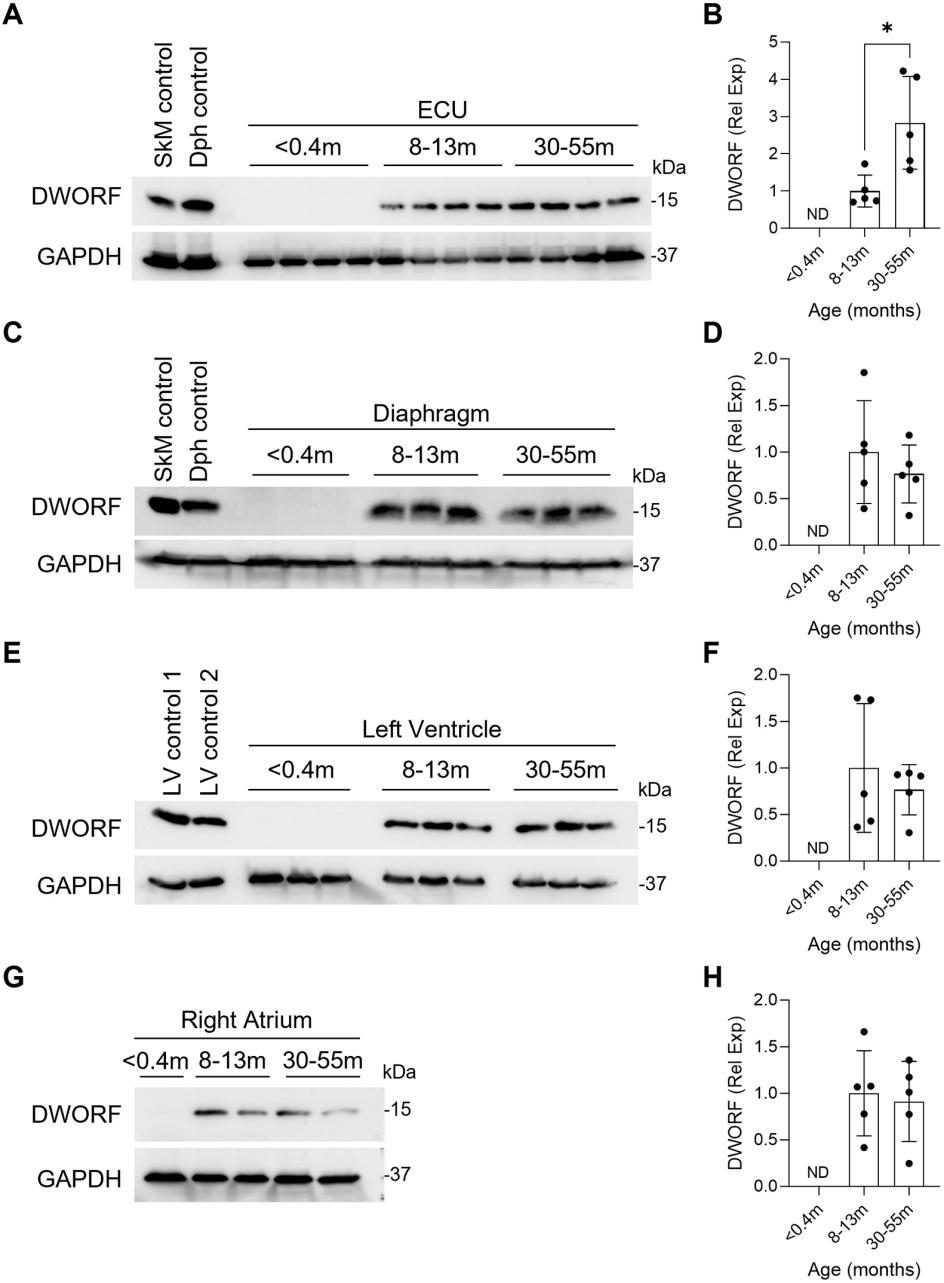
**DWORF expression in normal canine muscles from <0.4-month-old, 8- to 13-month-old and 30- to 55-month-old animals.** Western blot analysis performed on skeletal muscle and heart tissue from dogs in the following age groups: <0.4 months, 8-13 months and 30-55 months. (A,C,E,G) Representative western blots. (B,D,F,H) Quantification results performed by densitometry with normalization to GAPDH. All data are shown as relative to 8- to 13-month-old samples, where the average of the 8- to 13-month-old group is set to 1. Quantification panels included data from additional blots. (A,B) Extensor carpi ulnaris (ECU) muscle. *N*=5 for all groups. (C,D) Diaphragm muscle. *N*=5 for all groups. (E,F) Left ventricle (heart). *N*=5 for all groups. (G,H) Right atrium (heart). *N*=1 for <0.4-month-old and *N*=5 for 8- to 13-month-old and 30- to 55-month-old groups. Data are presented as mean±s.d. *P<0.05 (Wilcoxon rank-sum test). ND, not detected; Rel Exp, relative expression. Skeletal muscle (SkM) control was from the gastrocnemius muscle of a 13-month-old normal dog; diaphragm (Dph) control was from a 13-month-old normal dog; left ventricle (LV) control 1 was from a 12-month-old normal dog; LV control 2 was from a 13-month-old normal dog.

DWORF protein was undetectable in all tissues examined from neonatal dogs (Fig. 3). In contrast, robust DWORF expression was observed in the ECU (Fig. 3A,B), diaphragm (Fig. 3C,D), LV (Fig. 3E,F) and RA (Fig. 3G,H) of adult dogs (≥8 months of age). In the majority of tissues analyzed (diaphragm, LV, RA), DWORF expression was equivalent in the 8- to 13-month-old and 30- to 55-month-old groups, but in ECU, there was a statistically significant increase in DWORF expression between the 8- to 13-month-old and 30- to 55-month-old groups. These findings indicate that DWORF expression is developmentally regulated in dogs and is predominantly restricted to postweaning stages.

### DWORF expression is reduced in muscle of adult affected dogs

**Fig. 4.**
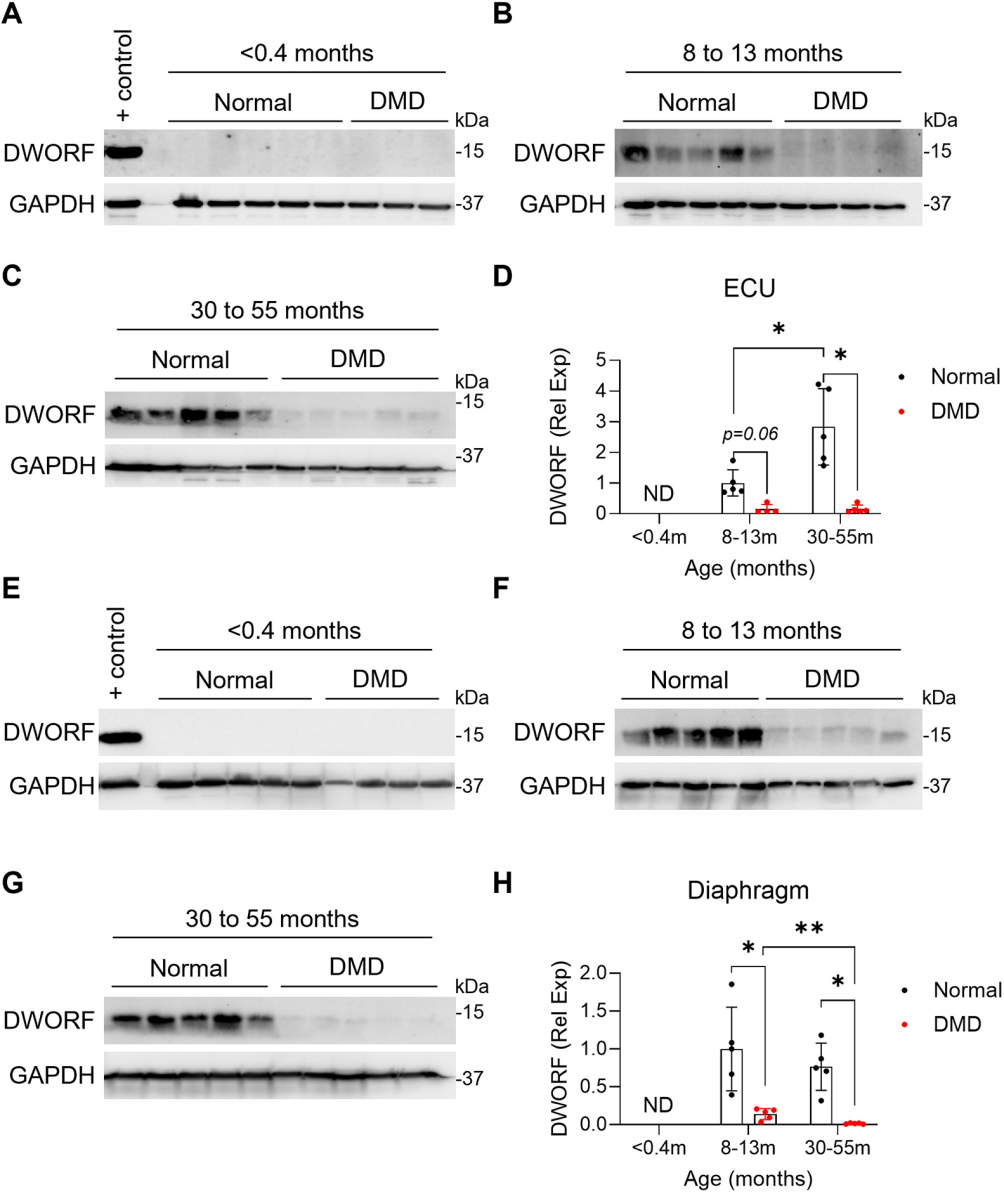
**DWORF expression in age-matched normal and affected canine skeletal muscles.** (A-H) Western blot analysis of DWORF expression and quantification in canine extensor carpi ulnaris (ECU) muscle (A-D) and diaphragm (E-H) from normal and affected dogs. The following age groups were examined: <0.4 months (A,E), 8-13 months (B,F) and 30-55 months (C,G). Western blots were quantified by densitometry, normalized to GAPDH and expressed as relative to 8- to 13-month-old normal dogs, where the average of the 8- to 13-month-old normal dogs is set to 1. (D,H) Sample sizes are as follows. ECU: <0.4-month-old, *N*=5 normal, *N*=3 DMD; 8 to 13-month-old, *N*=5 normal, *N*=4 DMD; 30 to 55-month-old, *N*=5 normal, *N*=5 DMD. Diaphragm: <0.4-month-old, *N*=5 normal, *N*=4 DMD; 8- to 13-month-old, *N*=5 normal, *N*=5 DMD; 30- to 55-month-old, *N*=5 normal, *N*=5 DMD. Data are presented as mean±s.d. *P<0.05; **P<0.01 (post-hoc pairwise Wilcoxon rank-sum test with Bonferroni correction). ND, not detected; Rel Exp, relative expression. The positive (+) control sample in A and E is from diaphragm muscle of a 13-month-old normal dog.

**Fig. 5.**
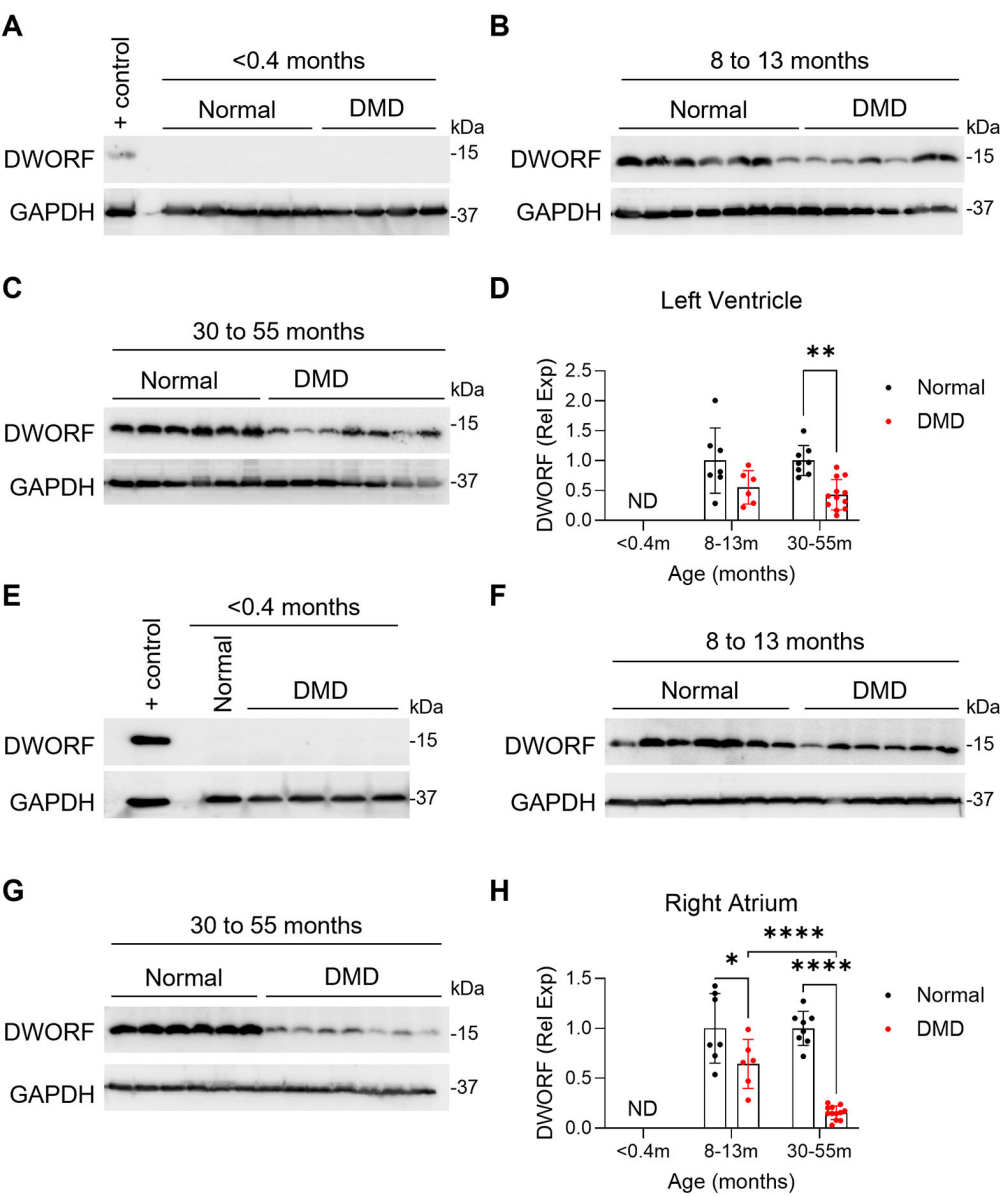
**DWORF expression in age-matched normal and affected canine heart tissue**. (A-H) Western blot analysis of DWORF expression and quantification in the left ventricle (A-D) and right atrium (E-H) from normal and affected dogs. The following age groups were examined: <0.4 months (A,E), 8-13 months (B,F) and 30-55 months (C,G). Western blots were quantified by densitometry, normalized to GAPDH, and expressed as relative to that in 8- to 13-month-old normal dogs, where the average of the 8- to 13-month-old normal dogs is set to 1 (D,H) Quantification panels included data from additional blots. Sample sizes are as follows. Left ventricle: <0.4-month-old, *N*=5 normal, *N*=4 DMD; 8- to 13-month-old, *N*=7 normal, *N*=6 DMD; 30- to 55-month-old, *N*=8 normal, *N*=12 DMD. Right atrium: <0.4-month-old, *N*=1 normal, *N*=4 DMD; 8- to 13-month-old, *N*=7 normal, *N*=6 DMD; 30- to 55-month-old, *N*=8 normal, *N*=12 DMD. Data are presented as mean±s.d. *P<0.05; **P<0.01; ****P<0.001 (post-hoc pairwise *t*-test using Tukey's honestly significant difference procedure). ND, not detected; Rel Exp, relative expression. The positive (+) control in A is from the left ventricle of a 12-month-old normal dog, and the+control in panel E is from the left ventricle of a 32-month-old normal dog.

To investigate whether DMD affects DWORF expression, we analyzed DWORF protein levels in skeletal and cardiac tissues from affected dogs. Western blot analysis was conducted on skeletal muscle tissues, including the ECU and diaphragm (Fig. 4), as well as cardiac tissues, including the LV and RA (Fig. 5), across three age groups: <0.4 months (pre-symptomatic stage), 8-13 months (symptomatic stage) and 30-55 months (terminal stage).

Similar to normal neonatal dogs, DWORF expression was not detected in skeletal muscle from neonatal affected (DMD) dogs (Fig. 4A,E). In adult dog skeletal muscle, DWORF expression was significantly reduced in the ECU (Fig. 4B-D) and diaphragm (Fig. 4F-H) of affected dogs at 30-55 months of age. DWORF expression was also reduced in the ECU (Fig. 4B,D) and diaphragm (Fig. 4F,H) of affected dogs at 8-13 months, although statistical significance was only achieved in the diaphragm. The decrease in DWORF protein levels was particularly pronounced in skeletal muscle samples from 30- to 55-month-old DMD dogs, with near-complete loss of DWORF expression observed in several samples (Fig. 4C,G). Notably, in the diaphragm, a muscle group that is severely affected by DMD pathology (Duan et al., 2021), DWORF expression was significantly reduced in 30- to 55-month-old affected dogs in comparison with 8- to 13-month-old affected dogs (Fig. 4F-H), suggesting a progressive decline in expression as the disease advances.

Similarly, DWORF expression was not detected in cardiac tissue of neonatal affected dogs (Fig. 5A,E). In adult dog cardiac tissues, DWORF expression was significantly reduced in both the LV and RA of 30- to 55-month-old affected DMD dogs compared to controls (Fig. 5B-D,F-H). In the RA, a significant reduction was also observed in the younger 8- to 13-month-old affected group, suggesting that downregulation begins early and worsens with disease progression (Fig. 5F-H). These findings further support a progressive decline in DWORF expression with advancing age and disease severity. The observed reductions are likely biologically meaningful given the essential role of DWORF in calcium regulation and contractile function.

Collectively, these findings indicate that DMD negatively impacts DWORF expression in both skeletal and cardiac muscle, with significant reductions occurring in all tissues examined in 30- to 55-month-old affected dogs as well as in the early stages of disease in the ECU muscle and RA (8- to 13-month-old affected dogs). The loss of DWORF in affected tissues may contribute to impaired muscle function and underscores its potential as a therapeutic target for dystrophic muscle.

## DISCUSSION

This study provides new insights into the regulation and expression of DWORF in canine skeletal and cardiac muscle in normal and DMD dogs. Using a newly developed polyclonal antibody capable of detecting the canine DWORF protein, we demonstrate that DWORF expression is developmentally regulated in dogs, with levels undetectable in neonatal dogs and becoming pronounced in skeletal and cardiac muscle tissues of adult dogs. Furthermore, we demonstrate that DWORF expression is significantly reduced in heart and skeletal muscles of DMD-affected dogs, with a particularly progressive decline observed in the diaphragm and RA as the disease advances.

The upregulation of DWORF in adult canine muscle mirrors postnatal observations in murine models (Nelson et al., 2016), in which DWORF plays a critical role in calcium regulation by counteracting phospholamban-mediated inhibition of SERCA2a to enhance cardiac contractility. This regulatory mechanism is essential for maintaining calcium homeostasis and proper muscle contractility. Our findings highlight the importance of DWORF in adult muscle function, as its absence in neonatal dogs suggests that alternative regulatory mechanisms compensate for calcium cycling needs during early development. Interestingly, a recent study reported detectable *DWORF* transcript levels in canine DMD models younger than 2 months of age (Morales et al., 2024), indicating potential post-translational mechanisms regulating DWORF expression. This observation aligns with prior findings in murine studies (Makarewich et al., 2018; Nelson et al., 2016). Further research is necessary to elucidate these regulatory mechanisms and to understand the functional importance of DWORF in adult canine muscle and cardiac tissues.

The pronounced loss of DWORF expression in affected skeletal muscle, particularly in the diaphragm, underscores its potential relevance in dystrophic pathology. The diaphragm is one of the most severely affected muscles in DMD (Duan et al., 2021), exhibiting progressive contractile dysfunction and fibrosis. The nearly complete loss of DWORF observed in the diaphragm of adult affected dogs aligns with the hypothesis that reduced calcium cycling efficiency exacerbates muscle weakness and damage in DMD. The progressive reduction in DWORF expression with advancing disease further suggests that DWORF downregulation contributes to the deteriorating muscle function characteristic of DMD. Importantly, we also observed significant reduction in DWORF expression in cardiac tissue, including the LV and RA, indicating that the heart is similarly affected. Given the critical role of DWORF in enhancing SERCA2a-mediated calcium uptake, its downregulation might compromise calcium handling and contribute to the development of cardiomyopathy in DMD. The progressive decline in DWORF expression from 8-13 months to 30-55 months in affected dogs further suggests that both skeletal and cardiac muscles experience a cumulative loss of calcium regulatory capacity as the disease advances.

The mechanisms underlying DWORF downregulation in DMD muscles remain unclear. Chronic inflammation, fibrosis and altered intracellular signaling in dystrophic muscle may suppress DWORF expression directly or indirectly; however, this has not been examined directly in our study. Our previous work across multiple heart failure models consistently showed a more pronounced reduction in DWORF protein levels compared to mRNA levels, suggesting that post-transcriptional or post-translational mechanisms play a significant role in regulating DWORF expression (Makarewich et al., 2020, 2018; Morales et al., 2024, 2023; Nelson et al., 2016). In the context of development, DWORF expression is undetectable in neonatal dogs and becomes prominent in both skeletal and cardiac muscle by 8-13 months of age, a pattern also observed in murine models. Future studies will be needed to define the molecular mechanisms governing DWORF regulation, including the potential roles of post-translational modifications or protein stability pathways, and to determine whether restoring DWORF expression in dystrophic muscle can enhance calcium cycling and mitigate disease progression.

Our findings also have significant therapeutic implications. DWORF enhances SERCA activity, promoting efficient calcium extrusion from the cytosol and improving overall calcium handling, making it an attractive target for therapeutic intervention in DMD. Strategies aimed at overexpressing DWORF, such as gene therapy approaches, are of high translational interest. Previous studies have shown that DWORF overexpression is cardioprotective in experimental and genetic models of disease, including the *mdx* mouse model of DMD (Morales et al., 2023). However, although the *mdx* mouse model has been used extensively in DMD studies, it fails to recapitulate several important features of the human disease (Yucel et al., 2018). *mdx* mice exhibit slow disease progression and near-normal lifespan, whereas patients with DMD have a mean life expectancy of 20.3 years (Landfeldt et al., 2020; McGreevy et al., 2015; Van Erp et al., 2010; Yucel et al., 2018). *mdx* mice also show only mild cardiac defects, and cardiac dysfunction is not evident until late in life (>12 months), with the phenotype being much more pronounced in female than male *mdx* mice (Bostick et al., 2010, 2009). Thus, the canine model, which effectively mimics the human disease with progressive muscle weakness, respiratory decline and severe cardiomyopathy (Duan, 2015; Kornegay, 2017; McGreevy et al., 2015), is a critical intermediary for preclinical evaluation of DWORF-targeted therapies.

In conclusion, this study establishes the postnatal expression pattern of DWORF protein in canine cardiac and skeletal muscle and demonstrates its progressive downregulation in the muscles of affected dogs. These findings provide a foundation for future studies investigating the role of DWORF in DMD pathophysiology and its potential as a therapeutic target. Furthermore, the newly developed anti-DWORF antibody described in this study not only detects the canine protein, but also cross-reacts with DWORF in human, cat, and pig tissues, making it a versatile tool for studying DWORF biology across multiple large-animal species. This broad utility is particularly valuable given the importance of dog, cat and pig models as a translational bridge between mouse and human. Importantly, this antibody will enable rigorous evaluation of DWORF expression in future AAV-based gene therapy studies in DMD dogs, thereby facilitating the translational development of DWORF-targeted interventions.

## MATERIALS AND METHODS

### Tissues

This study used curated tissues previously collected at the University of Missouri and Cincinnati Children's Hospital Medical Center. Animal tissue collection was approved by the Animal Care and Use Committee of the University of Missouri and Cincinnati Children's Hospital Medical Center. Specifically, mouse tissues were collected at Cincinnati Children's Hospital Medical Center from wild-type (*n*=1) or DWORF knockout (*n*=1) mice. Dog (*n*=43), cat (*n*=1), rabbit (*n*=1) and pig (*n*=1) tissues were collected from euthanized animals according to our published protocol at the University of Missouri (Hakim et al., 2023). Tissues from mixed-breed normal and dystrophin-null dogs were used in the study. The disease course of dystrophin-deficient dogs is well established (Kornegay, 2017; Kornegay et al., 2012; McGreevy et al., 2015). Neonatal affected dogs show nominal muscle disease. Eight- to 13-month-old affected dogs display characteristic clinical manifestations of muscular dystrophy. Affected dogs typically die by 3-4 years of age. Dog tissues used in this study were selected to reflect different stages of the disease course, including pre-symptomatic (<0.4 months), symptomatic (8 to 13 months) and terminal (30-55 months). No animals were euthanized for the purpose of this study. Experimental dog information is provided in Table 1, with each sample detailed in Table S1. Human tissue collection was approved by the University of Missouri Institutional Review Board and conducted according to the principles expressed in the Declaration of Helsinki. A de-identified human muscle tissue was obtained from a lower limb amputation performed at the University of Missouri Hospital via the University of Missouri OneHealth Biorepository.

### Production of canine-specific DWORF polyclonal antibody

The affinity-purified rabbit anti-dog DWORF was manufactured by Vivitide (Gardner, MA, USA) using a customer-designed peptide H2N-MAEKAALSNLLCKK amide. The region of ‘MAEKAALSNLL’ was the N-terminal end of dog DWORF. ‘C’ was added for conjugation. ‘KK’ was added to improve solubility. Specifically, two New Zealand white rabbits were immunized in a specific pathogen-free facility. Serum was collected before and after peptide immunization. Anti-dog DWORF polyclonal antibody was purified from serum using an affinity column.

### Western blotting

Freshly isolated skeletal muscle and heart tissues were snap frozen in liquid nitrogen. Frozen tissues were homogenized with a glass dounce homogenizer in radioimmunoprecipitation assay (RIPA) buffer [150 mM NaCl; 1% v/v Igepal CA-630 (NP-40); 50 mM Tris-HCl, pH 8.0; 0.5% w/v sodium deoxycholate; 0.1% w/v sodium dodecyl sulfate, Sigma-Aldrich, St Louis, MO, USA] with added protease inhibitors (cOmplete ULTRA mini-tablet, Roche, Indianapolis, IN, USA) and phosphatase inhibitors (PhosSTOP tablet, Roche). Protein concentrations were determined by standard BCA protein assay (Pierce, Rockford, IL, USA). 30 μg of protein was run on 15% bis/acrylamide gels made by standard preparation, and proteins were transferred to PVDF membranes (Immoblion-P, Millipore, Waltham, MA, USA) by wet transfer technique (20% methanol). Membranes were blocked with 5% bovine serum albumin (BSA; Sigma-Aldrich) in Tris-buffered saline (TBS) with 1% Tween-20 (TBST) for 30 min at room temperature, then incubated overnight at 4°C with rabbit polyclonal antibody against canine DWORF generated in this study at a concentration of 1:2000 or a previously validated custom rabbit polyclonal antibody against mouse DWORF (New England Peptide, Gardner, MA, USA) at a concentration of 1:1000 (Nelson et al., 2016). Blots were washed with TBST, incubated with a goat anti-rabbit IgG (H+L)-HRP conjugate secondary antibody (Bio-Rad, Hercules, CA, USA) and developed using Clarity™ Western Enhanced Chemiluminescence (ECL) substrate (Bio-Rad). Images were acquired using a Bio-Rad ChemiDoc MP Imaging System using signal acquisition mode. Membranes were subsequently stripped with Restore™ PLUS Western Blot Stripping Buffer (Thermo Fisher Scientific, Waltham, MA, USA), re-blocked, and incubated in mouse monoclonal anti-GAPDH antibody (Millipore) diluted 1:5000 overnight at 4°C. Blots were washed with TBST, incubated with a goat anti-mouse IgG (H+L)-HRP conjugate secondary antibody (Bio-Rad), developed and imaged as described above. Western blots were quantified using ImageLab 6.1 Software (Bio-Rad). Internal controls were used on all western blots to maintain consistency across individual blots. All uncropped western blots are included in Fig. S1.

### Multiple sequence alignment

Protein sequences were aligned using the European Molecular Biology Laboratory - European Bioinformatics Institute (EMBL-EBI) Clustal Omega (Madeira et al., 2024). Sequences in FASTA format were uploaded to the Clustal Omega web server (https://www.ebi.ac.uk/jdispatcher/msa/clustalo) and default parameters were used for alignment.

### Statistical analysis

Data are presented as mean±s.d. Statistical analysis was performed using the Matlab Statistics and Machine Learning Toolbox (V24.1; Matlab 2024a, MathWorks Inc., Natick, MA, USA). The normality of the data was assessed using the Anderson–Darling test. For DWORF data that failed to meet the normality assumption, the non-parametric Kruskal–Wallis test was employed to examine the effects of age (<0.4 months, 8-13 months and 30-55 months) and muscle (LV, RA, diaphragm and ECU). Post-hoc pairwise comparisons were carried out using the Wilcoxon rank-sum test with Bonferroni correction. Additionally, the Wilcoxon rank-sum test was utilized to evaluate the statistical differences between normal and affected dogs. For data with normal distributions, two-way ANOVA was applied to study the effects of age and group, followed by pairwise comparison using Tukey's honestly significant difference procedure. *P*<0.05 was considered statistically significant.

## Supplementary Material

10.1242/dmm.052285_sup1 Supplementary information
