# Supplementary material for: DWORF expression is reduced in a large animal model of Duchenne muscular dystrophy
Source: Dis Model Mech. 2025 Jun 25;18(6):dmm052285. doi: 10.1242/dmm.052285 (PMC12233064; doi:10.1242/dmm.052285)
Supplement: Supplementary information [file dmm-18-052285-s1.pdf]

Fig. S1. Uncropped western blots used in Figs 1-5.

Notes related to all uncropped Western blots:

-Blots contain sample identifiers (IDs) for each tissue used, with corresponding IDs indicated in the Supplemental Table

-Samples in grey text were omitted from final figures

Uncropped Western blots corresponding to Figure 1

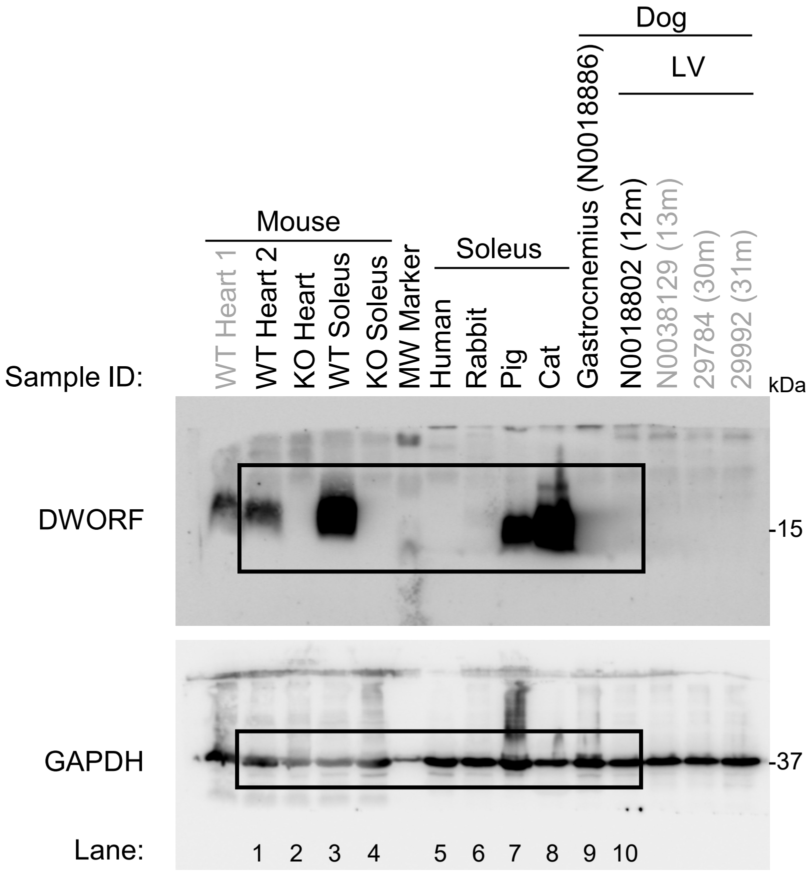

Disease Models & Mechanisms • Supplementary information

Uncropped Western blots corresponding to Figure 2B

Pre-immunization serum

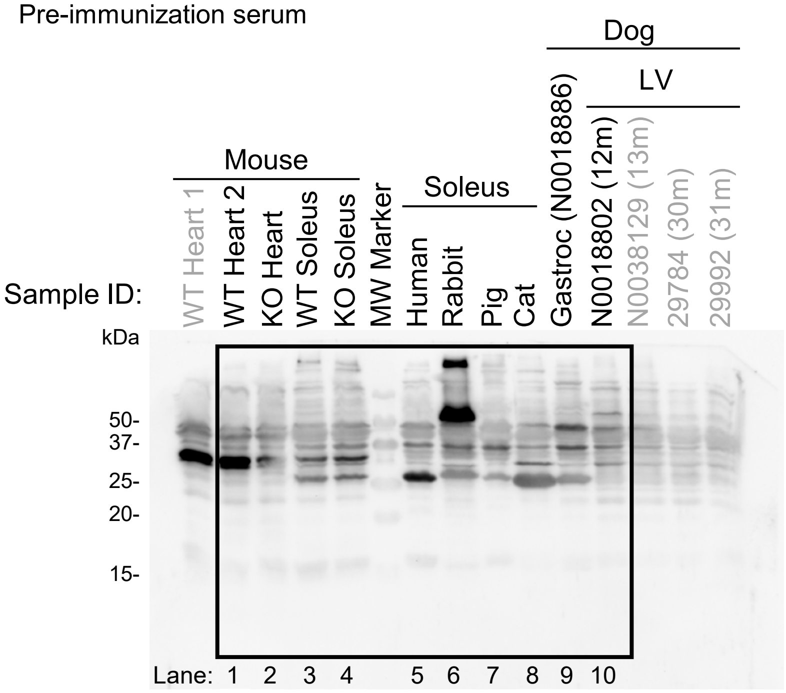

Uncropped Western blots corresponding to Figure 2C

Purified DWORF antibody

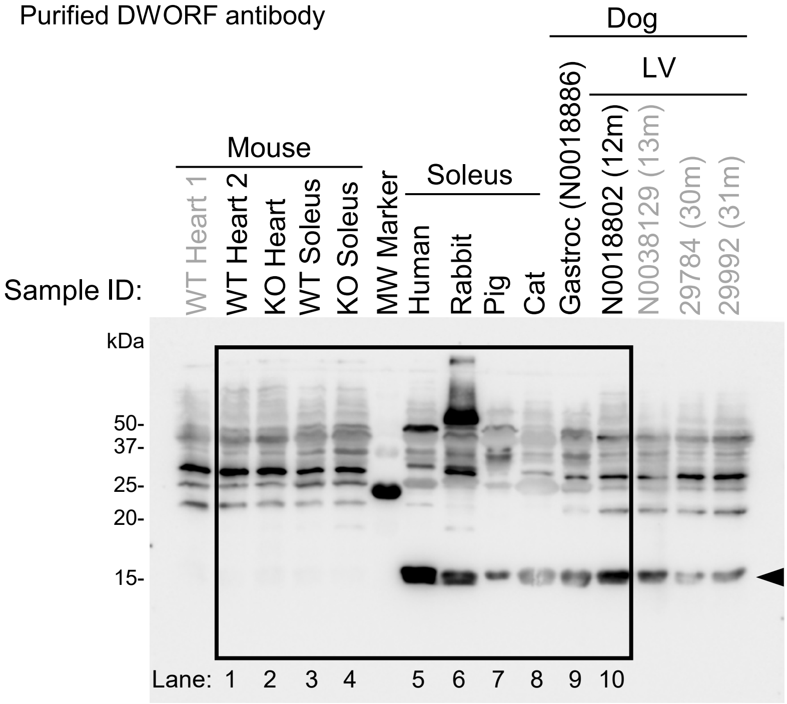

Disease Models & Mechanisms • Supplementary information

Uncropped Western blots corresponding to Figure 3A

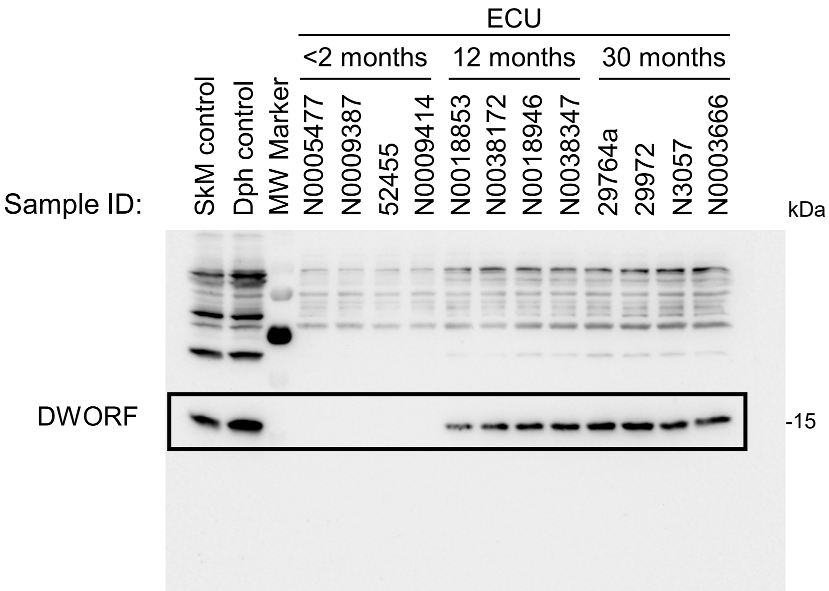

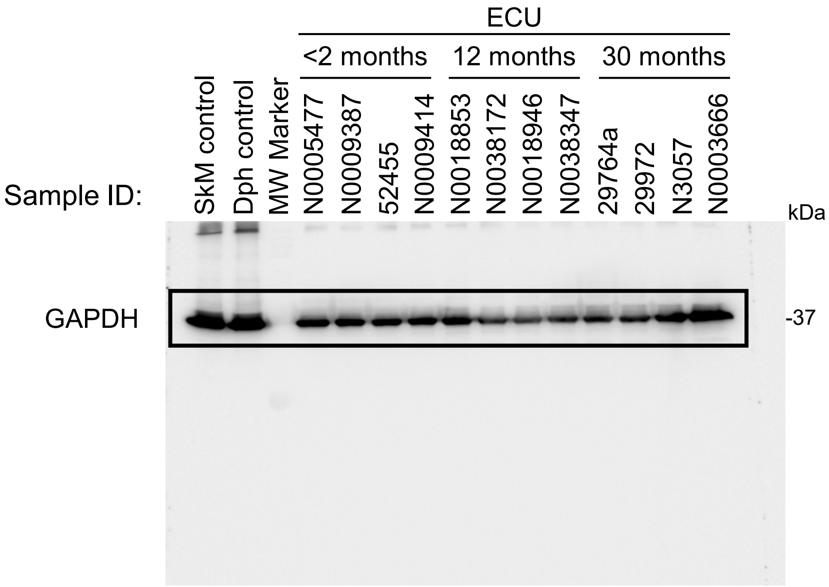

Uncropped Western blots corresponding to Figure 3C

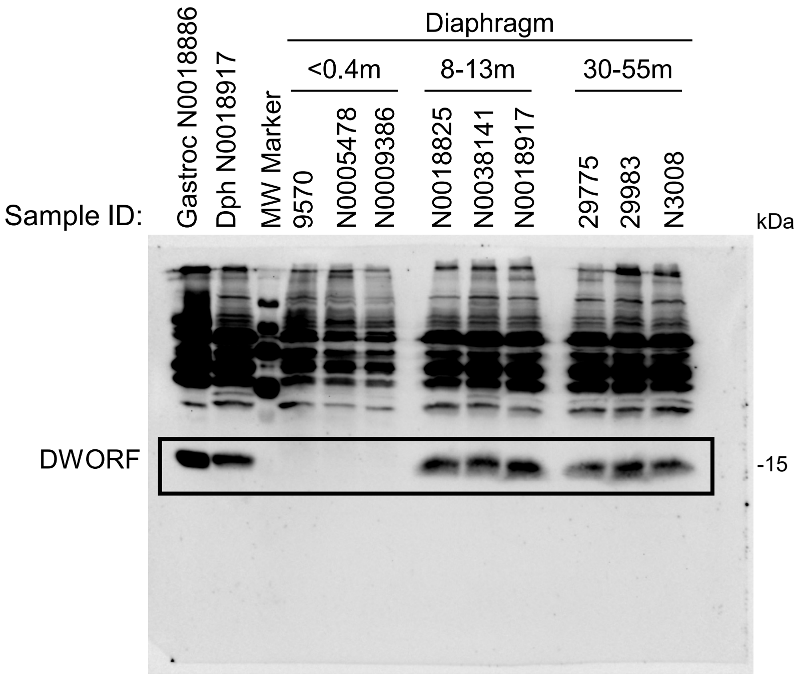

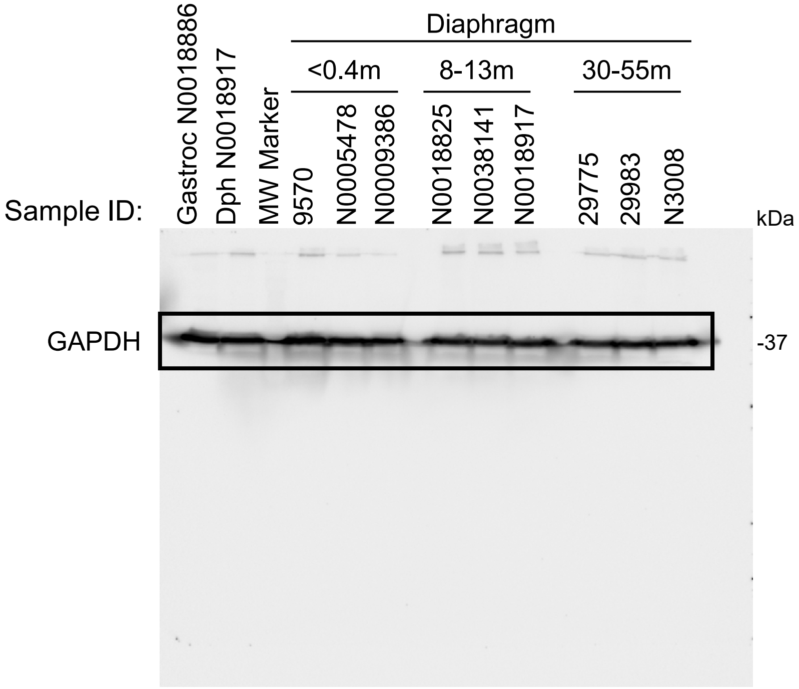

Uncropped Western blots corresponding to Figure 3E

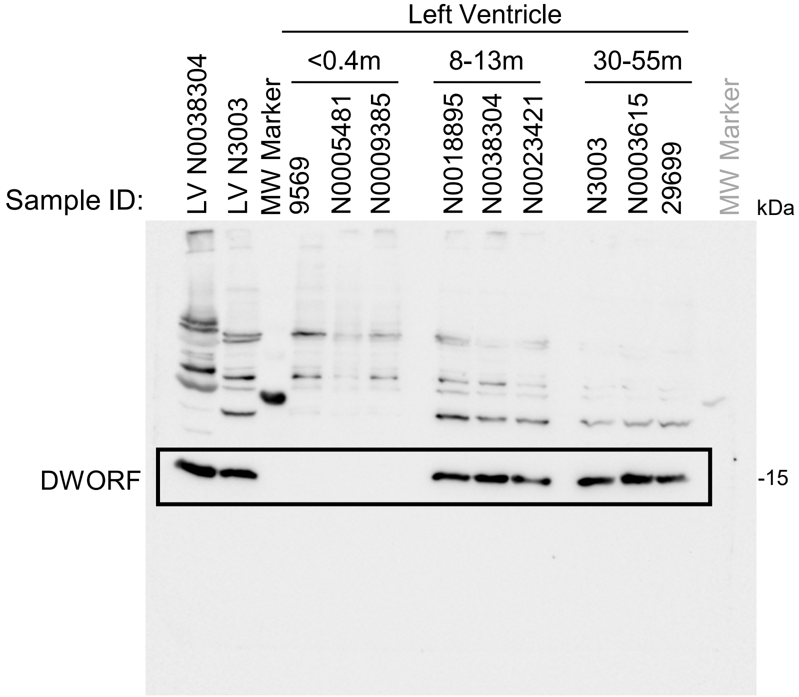

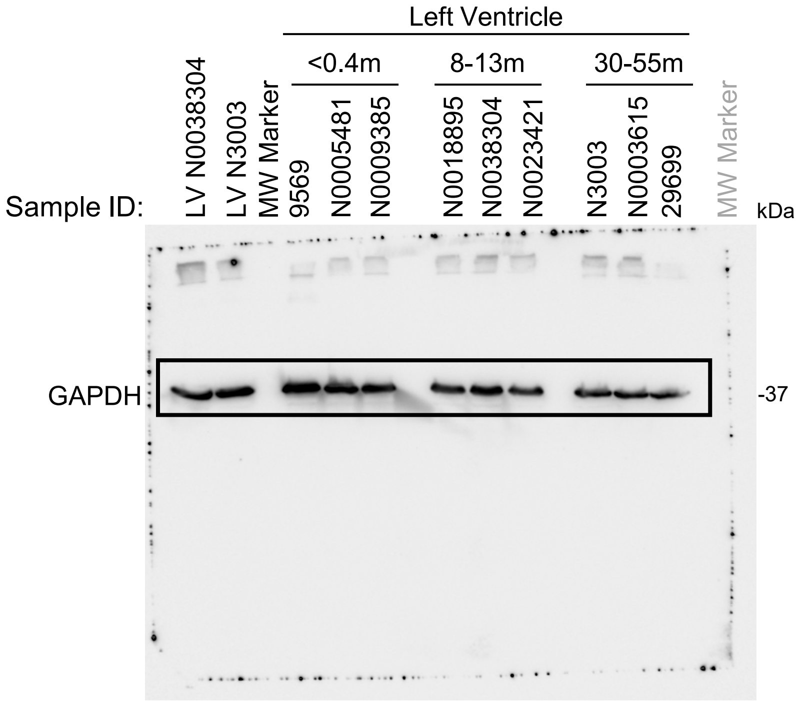

Uncropped Western blots corresponding to Figure 3G

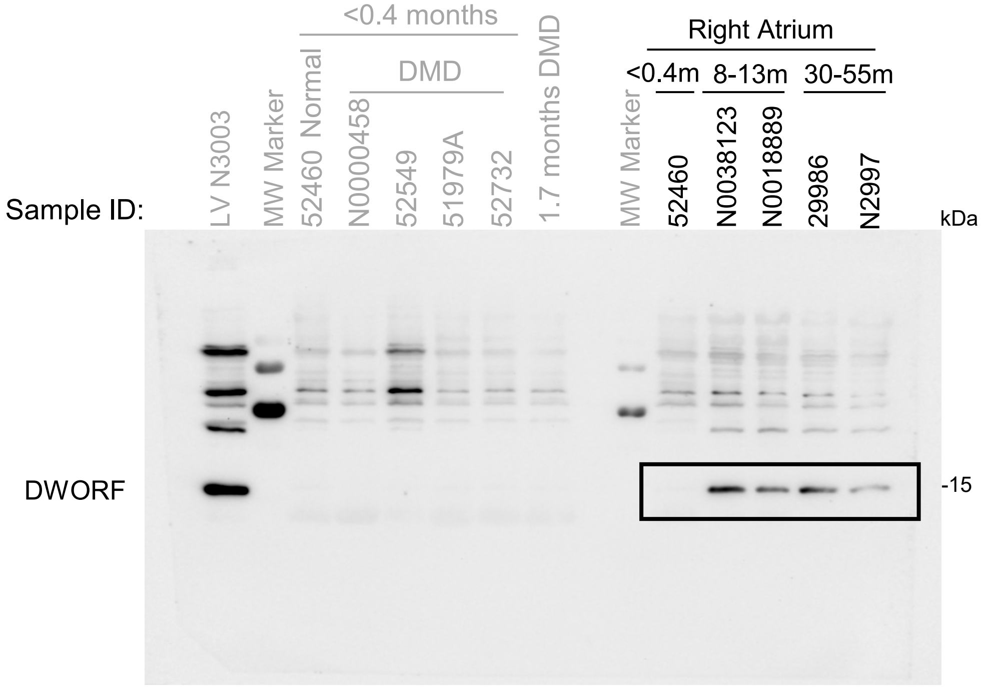

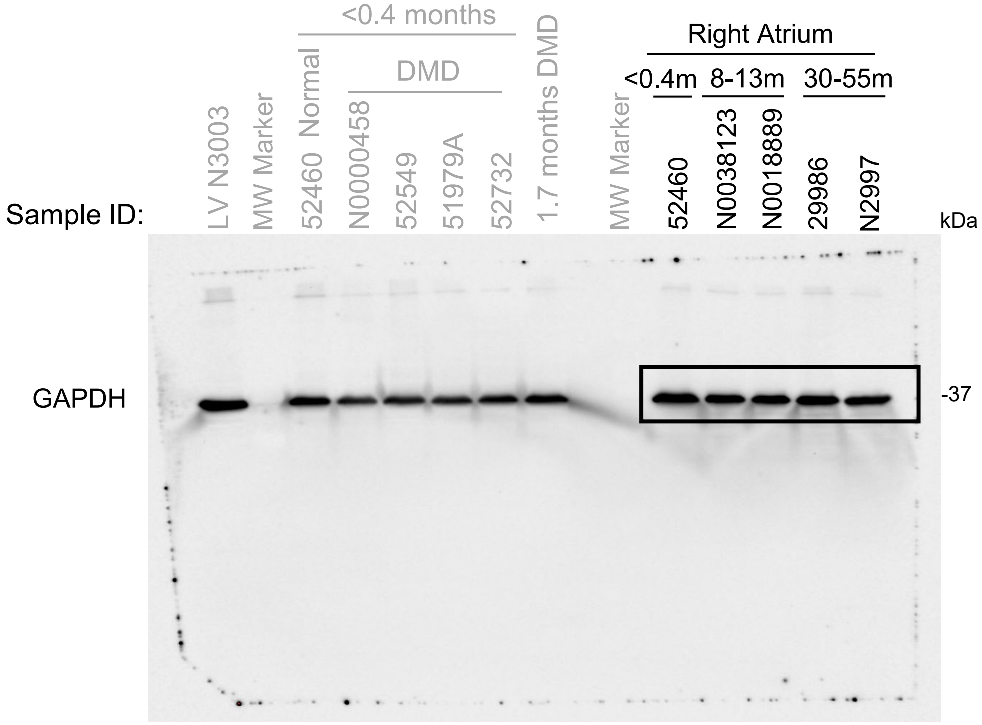

Disease Models & Mechanisms • Supplementary information

Uncropped Western blots corresponding to Figure 4A

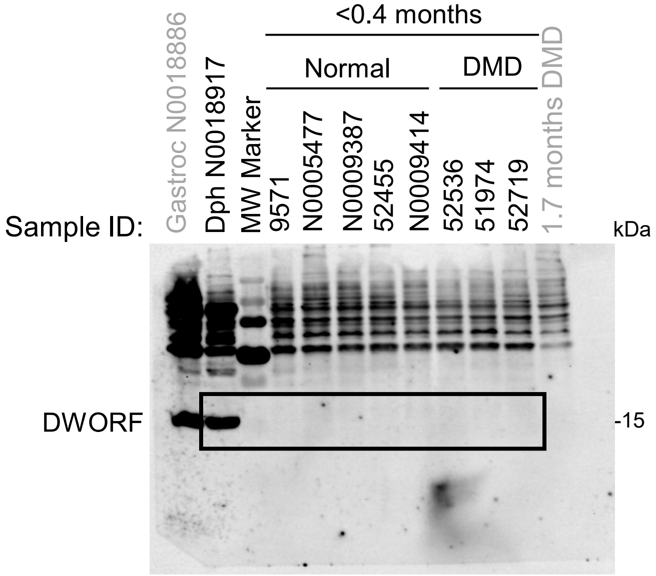

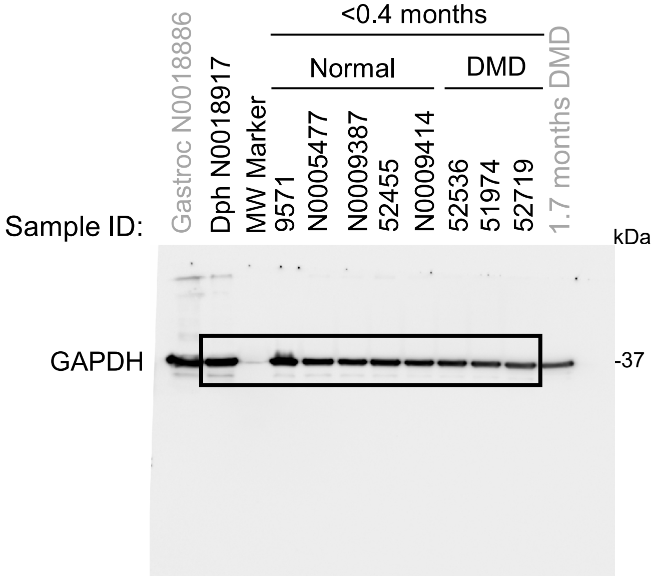

Uncropped Western blots corresponding to Figure 4B

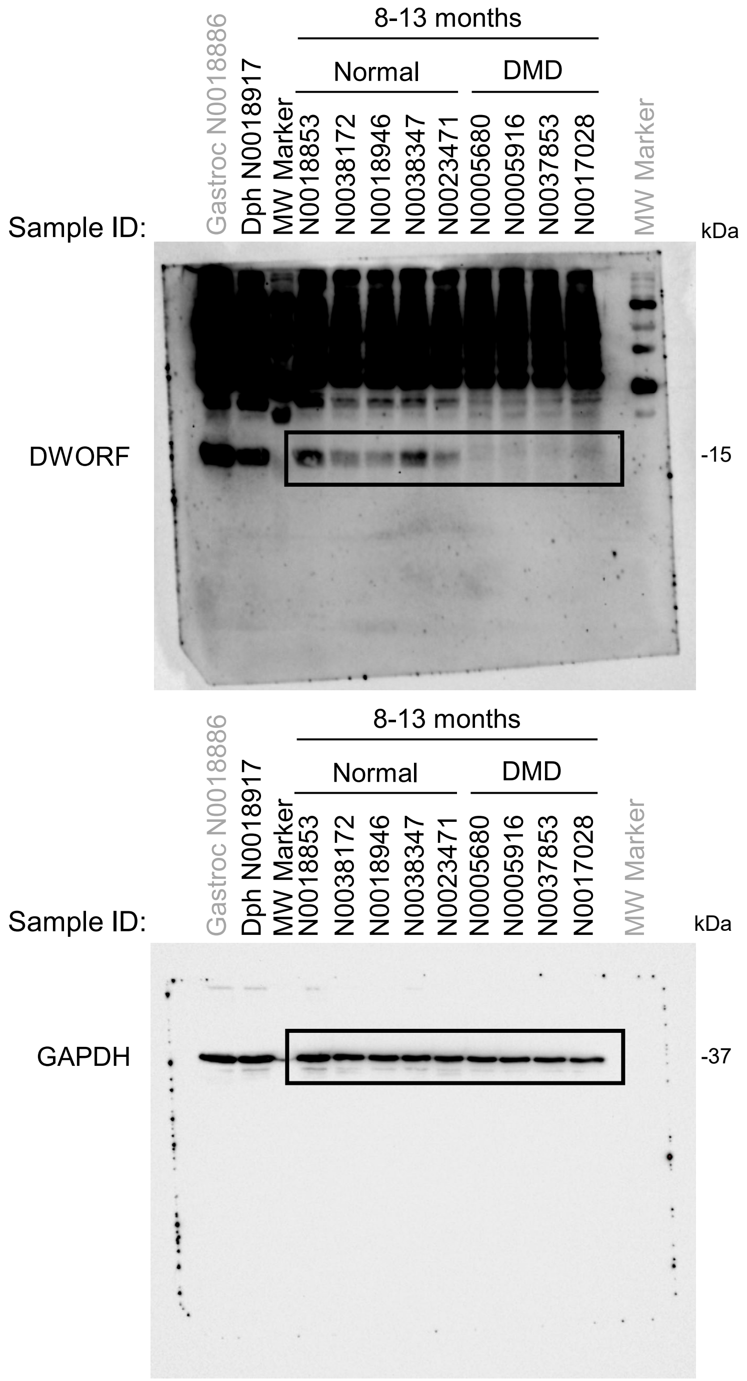

Disease Models & Mechanisms • Supplementary information

Uncropped Western blots corresponding to Figure 4C

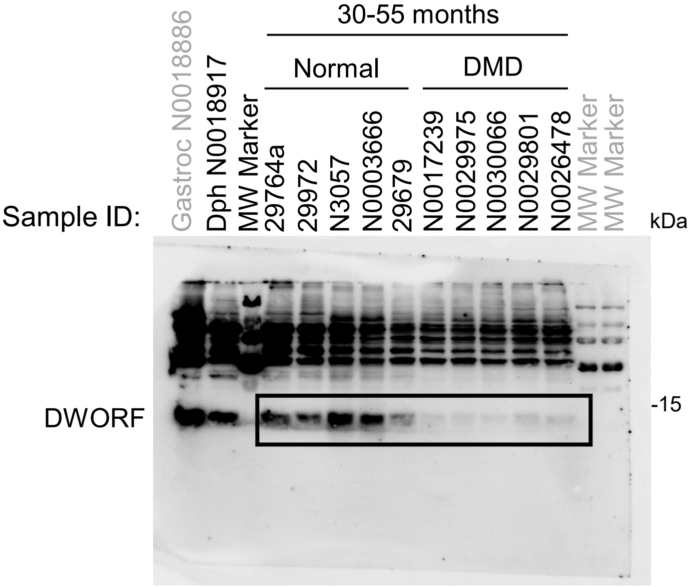

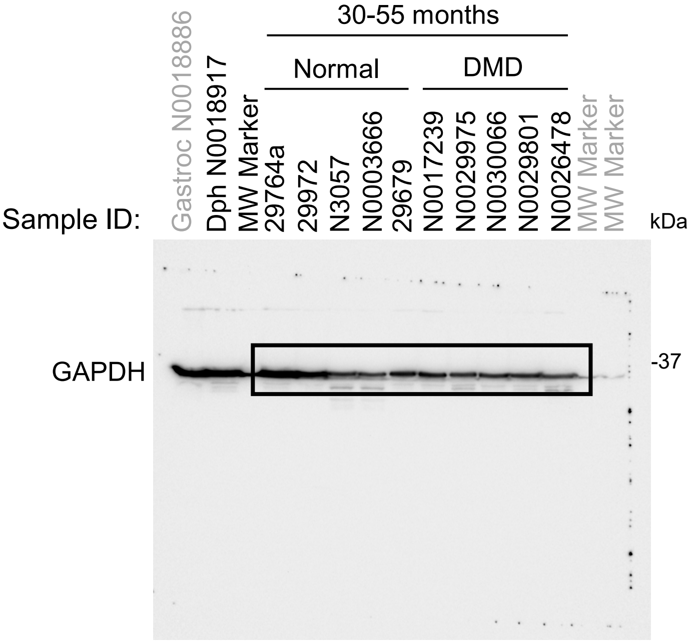

Uncropped Western blots corresponding to Figure 4E

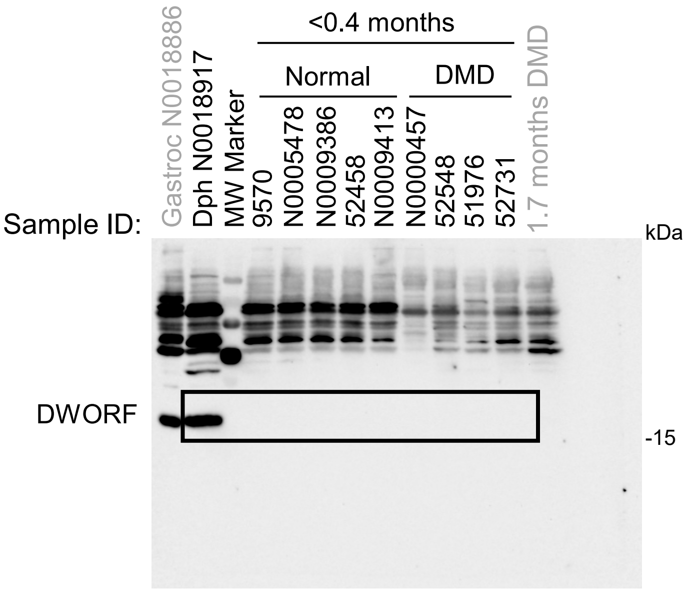

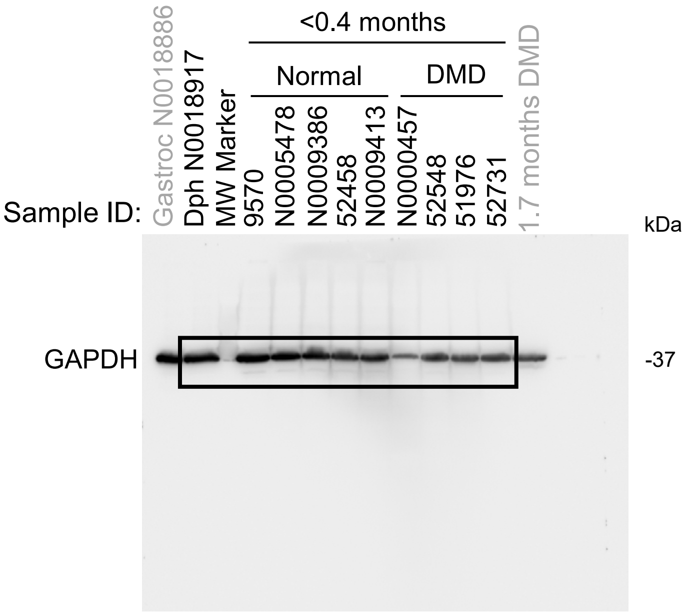

Uncropped Western blots corresponding to Figure 4F

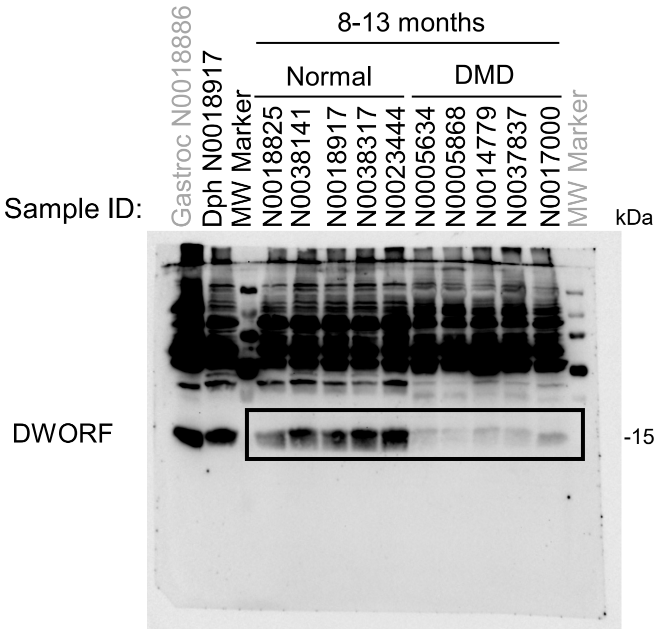

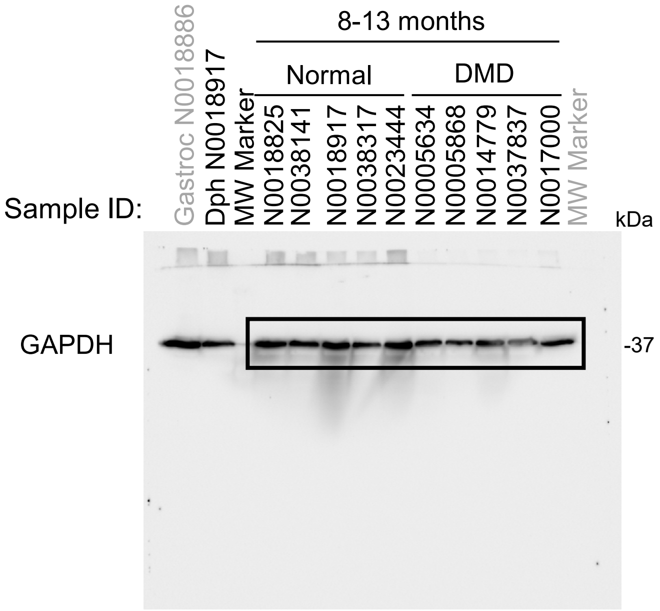

Uncropped Western blots corresponding to Figure 4G

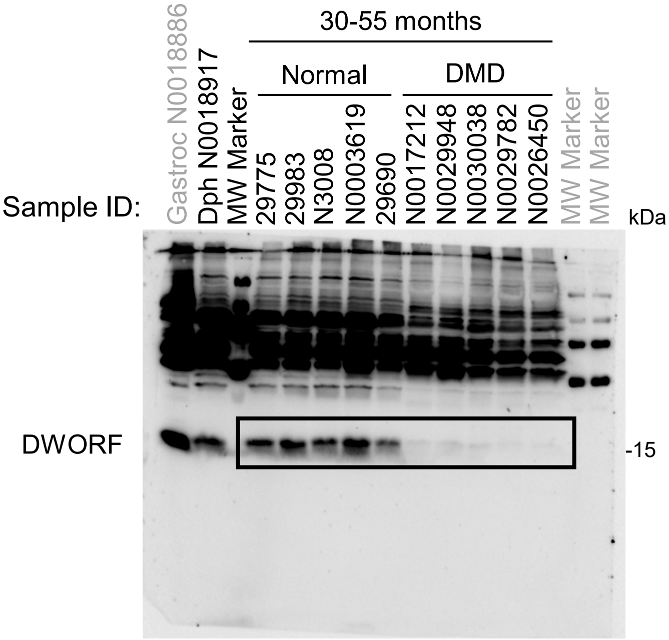

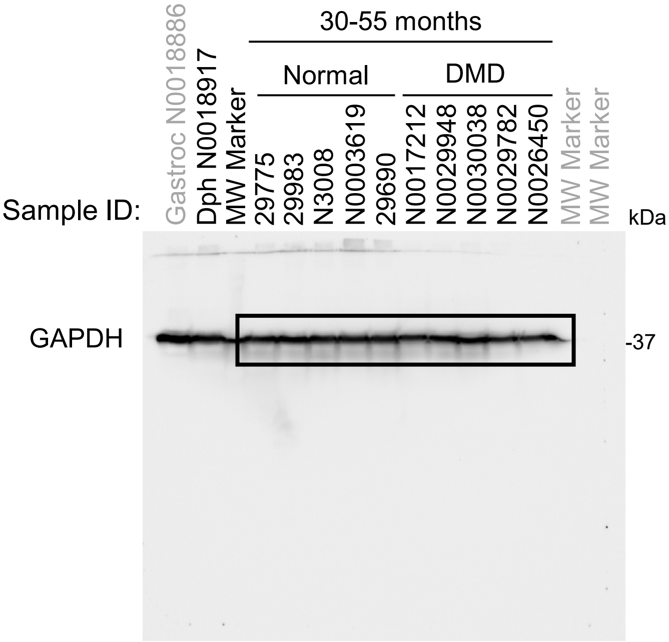

Disease Models & Mechanisms • Supplementary information

Uncropped Western blots corresponding to Figure 5A

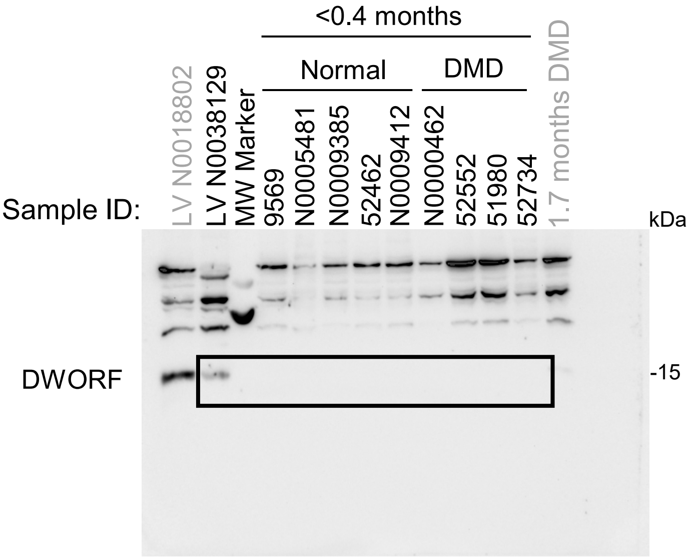

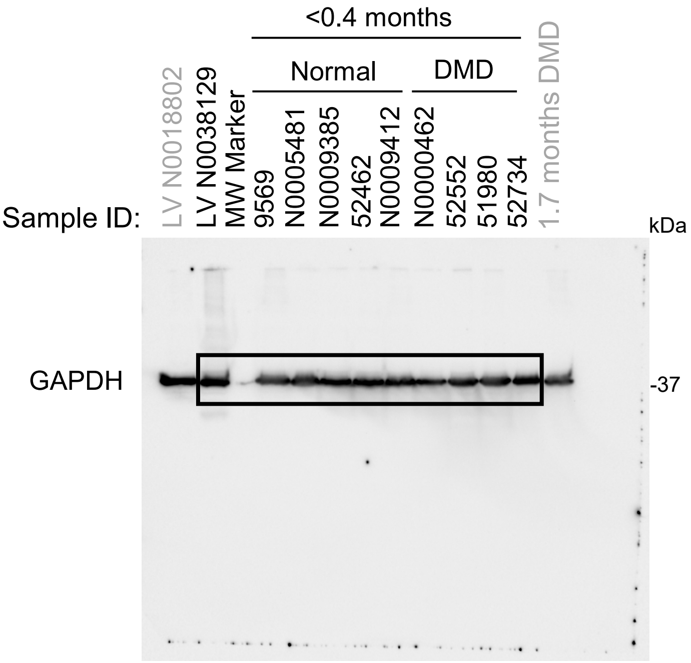

Uncropped Western blots corresponding to Figure 5B

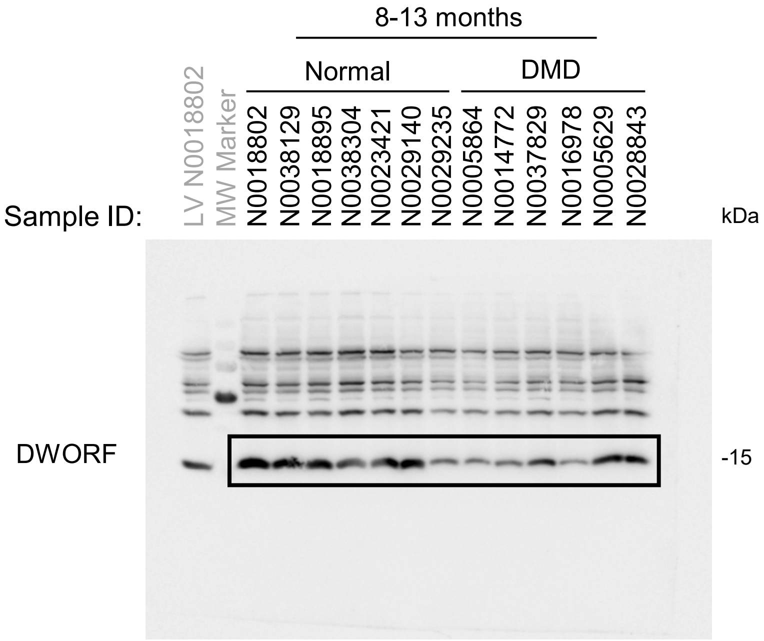

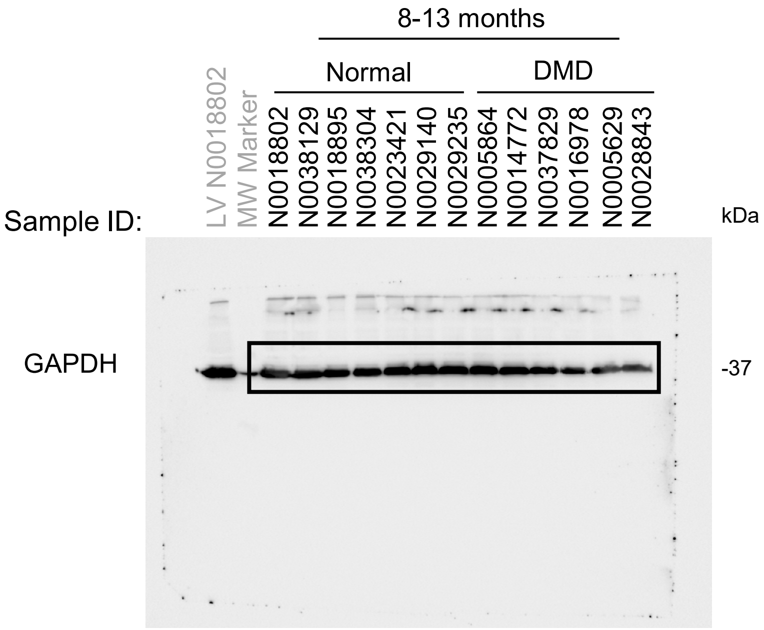

Uncropped Western blots corresponding to Figure 5C

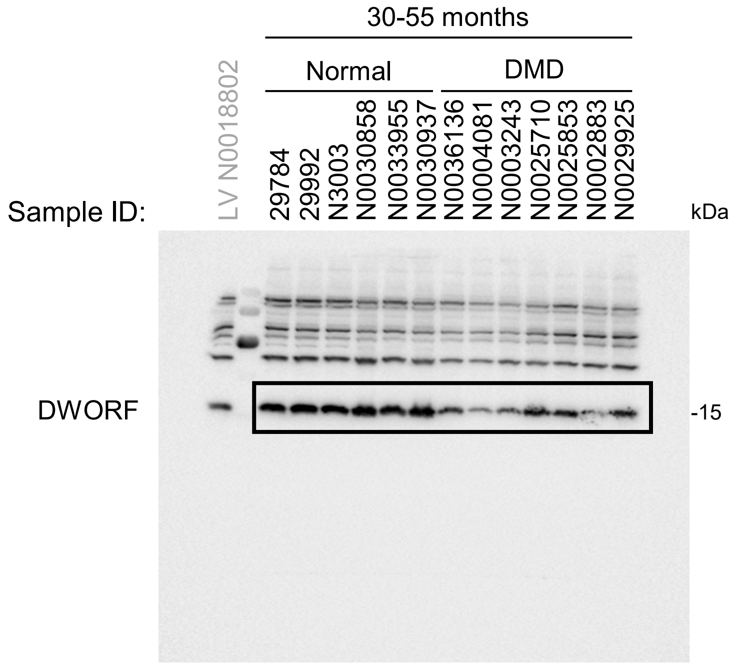

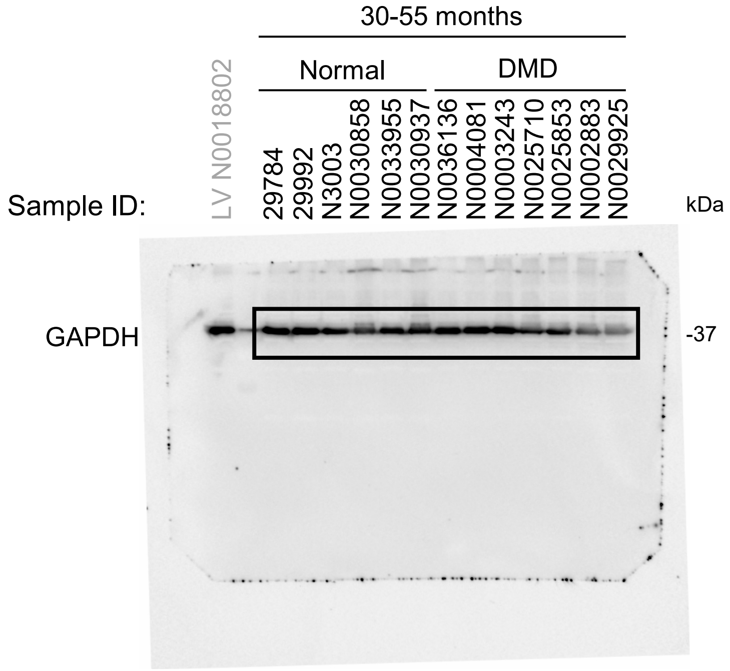

Disease Models & Mechanisms • Supplementary information

Uncropped Western blots corresponding to Figure 5E

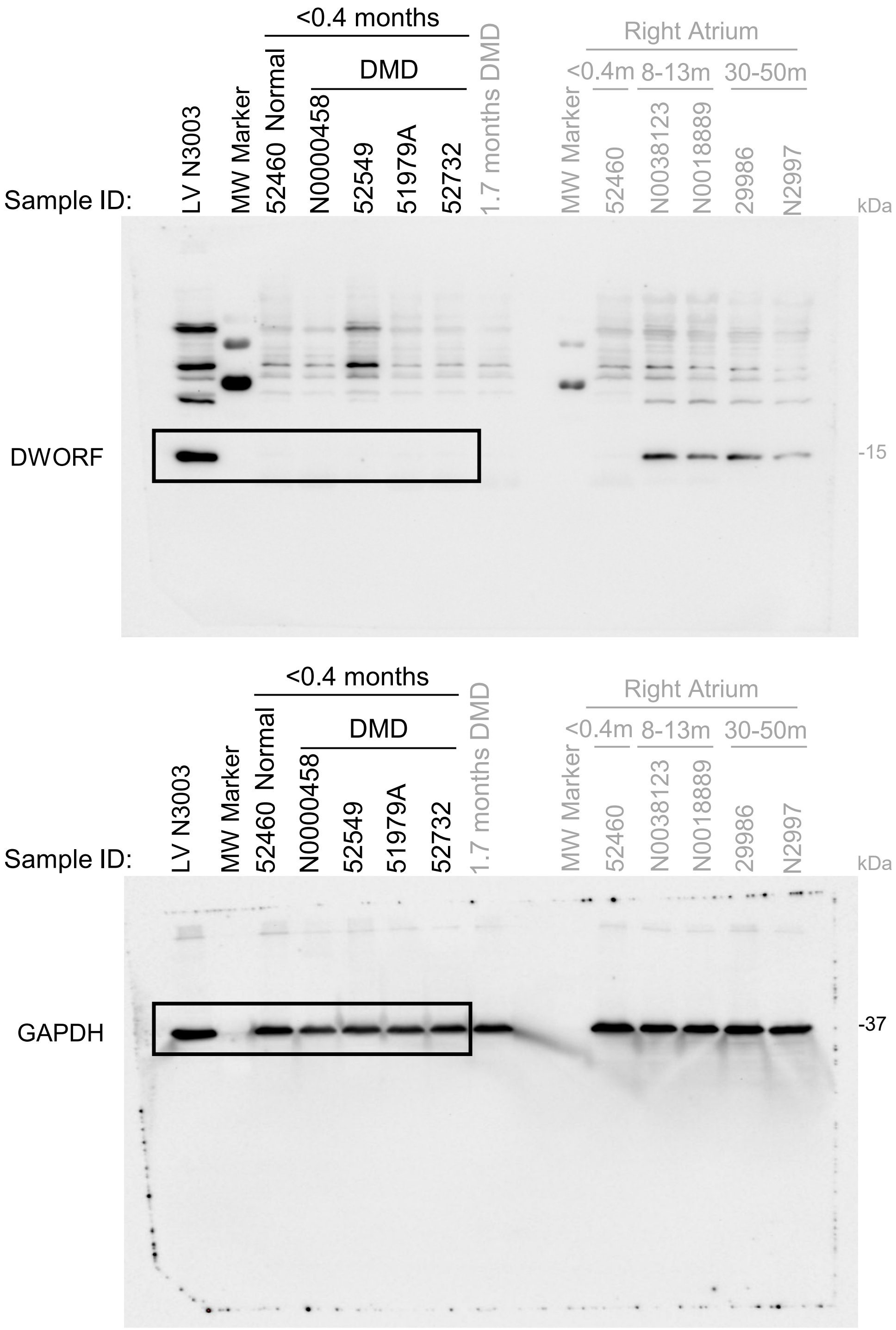

Uncropped Western blots corresponding to Figure 5F

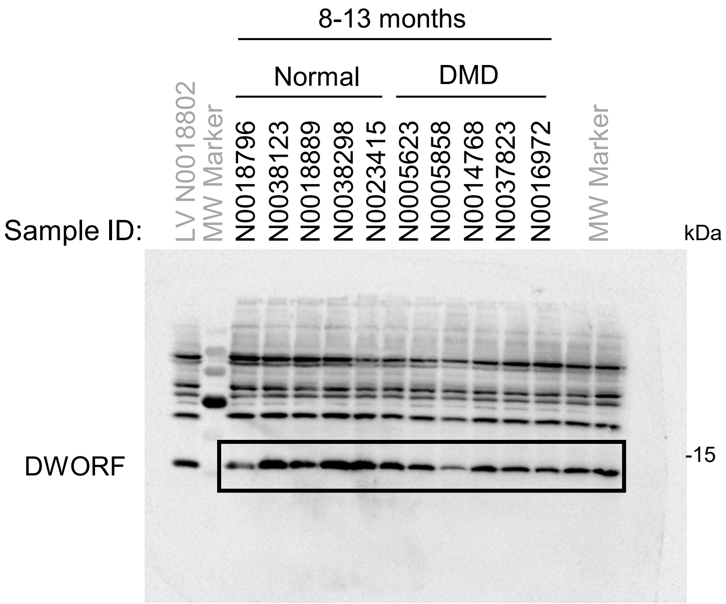

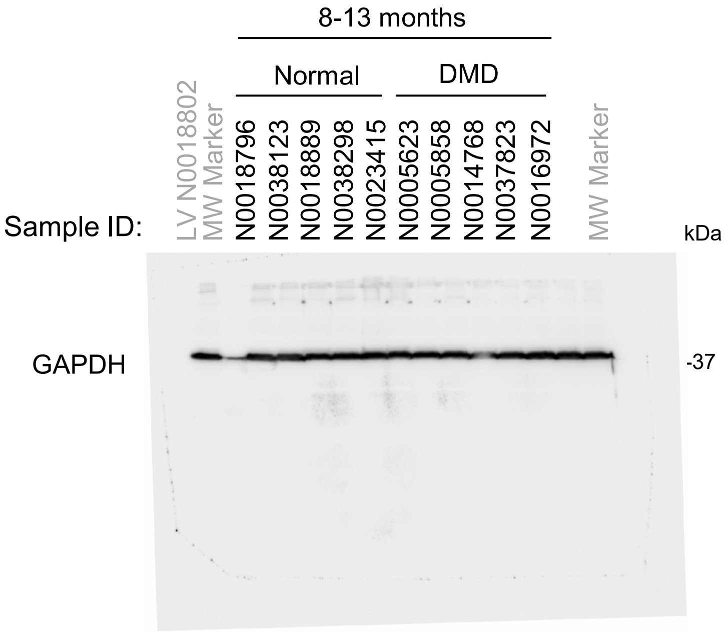

Disease Models & Mechanisms • Supplementary information

Uncropped Western blots corresponding to Figure 5G

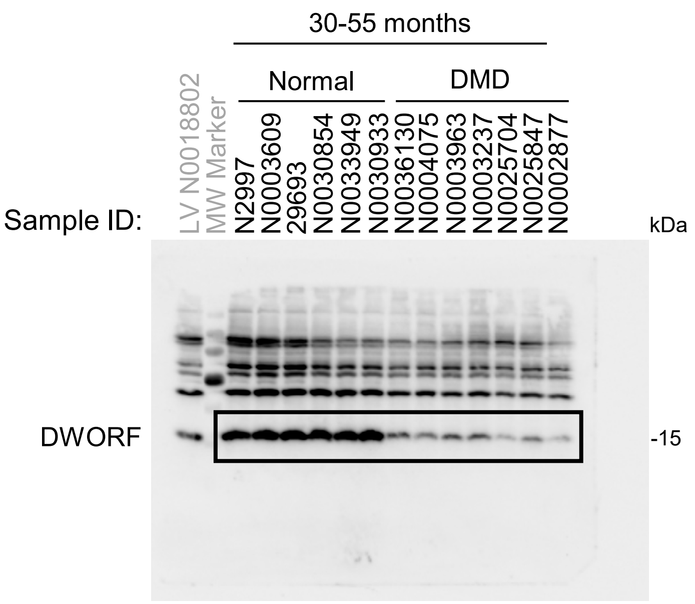

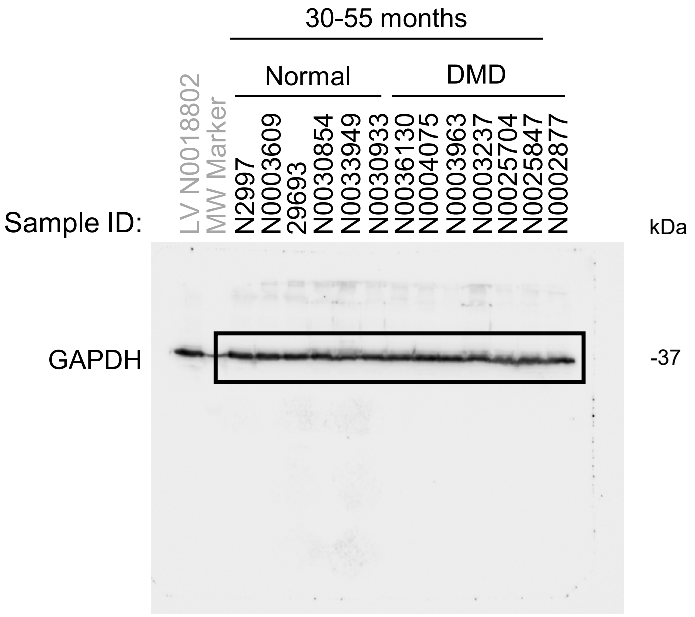

Disease Models & Mechanisms • Supplementary information

Table S1. Dog information with sample identifiers (IDs).

Age range	Dog ID#	Genotype	Sex	Age (m)	RA	LV	Diaphragm	ECU	Gas
< 0.4-month-old	Dog #1	Normal	M	0.03	No	9569	9570	9571	No
	Dog #2	Normal	M	0.07	No	N0005481	N0005478	N0005477	No
	Dog #3	Normal	M	0.13	No	N0009385	N0009386	N0009387	No
	Dog #4	Normal	M	0.17	52460	52462	52458	52455	No
	Dog #5	Normal	M	0.23	No	N0009412	N0009413	N0009414	No
	Dog #6	Affected	M	0.03	N0000458	N0000462	N0000457	No	No
	Dog #7	Affected	M	0.16	52549	52552	52548	52536	No
	Dog #8	Affected	F	0.30	51979A	51980	51976	51974	No
	Dog #9	Affected	M	0.36	52732	52734	52731	52719	No
8 to 13-month-old	Dog #10	Normal	F	8.61	N0029134	N0029140	No	No	No
	Dog #11	Normal	F	8.71	N0029229	N0029235	No	No	No
	Dog #12	Normal	M	12.30	N0018796	N0018802	N0018825	N0018853	N0018886
	Dog #13	Normal	M	12.56	N0038123	N0038129	N0038141	N0038172	No
	Dog #14	Normal	M	12.76	N0018889	N0018895	N0018917	N0018946	No
	Dog #15	Normal	M	12.92	N0038298	N0038304	N0038317	N0038347	No
	Dog #16	Normal	M	13.28	N0023415	N0023421	N0023444	N0023471	No
	Dog #17	Affected	M	8.02	N0028837	N0028843	No	No	No
	Dog #18	Affected	F	8.78	N0005623	N0005629	N0005634	N0005680	No
	Dog #19	Affected	M	8.91	N0005858	N0005864	N0005868	N0005916	No
	Dog #20	Affected	F	8.98	N0014768	N0014772	N0014779	No	No
	Dog #21	Affected	M	12.46	N0037823	N0037829	N0037837	N0037853	No
	Dog #22	Affected	M	13.05	N0016972	N0016978	N0017000	N0017028	No
30 to 55-month-old	Dog #23	Normal	M	29.92	29778	29784	29775	29764a	No
	Dog #24	Normal	M	30.61	29986	29992	29983	29972	No
	Dog #25	Normal	M	31.50	N2997	N3003	N3008	N3057	No
	Dog #26	Normal	M	36.82	N0030933	N0030937	No	No	No
	Dog #27	Normal	M	37.38	N0030854	N0030858	No	No	No
	Dog #28	Normal	M	45.47	N0003609	N0003615	N0003619	N0003666	No
	Dog #29	Normal	M	47.84	N0033949	N0033955	No	No	No
	Dog #30	Normal	F	55.43	29693	29699	29690	29679	No
	Dog #31	Affected	F	30.81	N0003963	No	No	No	No
	Dog #32	Affected	F	30.84	N0004075	N0004081	No	No	No
	Dog #33	Affected	M	33.04	N0003237	N0003243	No	No	No
	Dog #34	Affected	M	33.30	N0017183	N0017189	N0017212	N0017239	No
	Dog #35	Affected	M	34.26	N0029768	N0029774	N0029782	N0029801	No
	Dog #36	Affected	M	34.82	N0029919	N0029926	N0029948	N0029975	No
	Dog #37	Affected	M	38.70	N0030010	N0030016	N0030038	N0030066	No
	Dog #38	Affected	M	38.70	No	N0029925	No	No	No
	Dog #39	Affected	M	41.62	N0025704	N0025710	No	No	No
	Dog #40	Affected	M	41.79	N0002877	N0002883	No	No	No
	Dog #41	Affected	M	41.95	N0025847	N0025853	No	No	No
	Dog #42	Affected	M	44.81	N0026422	N0026428	N0026450	N0026478	No
	Dog #43	Affected	M	46.06	N0036130	N0036136	No	No	No

Abbreviations: ECU, extensor carpi ulnaris; Gas, gastrocnemius; LV, left ventricle; RA, right atrium.
